# Rational Design of Rod‐Like Liquid Crystals Exhibiting Two Nematic Phases

**DOI:** 10.1002/chem.201702742

**Published:** 2017-09-18

**Authors:** Richard J. Mandle, Stephen J. Cowling, John W. Goodby

**Affiliations:** ^1^ Department of Chemistry University of York York YO10 5DD UK

**Keywords:** density functional calculations, liquid crystals, soft matter, X-ray scattering

## Abstract

Recently, a polar, rod‐like liquid‐crystalline material was reported to exhibit two distinct nematic mesophases (termed N and N_*X*_) separated by a weakly first‐order transition. Herein, we present our initial studies into the structure–property relationships that underpin the occurrence of the lower‐temperature nematic phase, and report several new materials that exhibit this same transformation. We have prepared material with significantly enhanced temperature ranges, allowing us to perform a detailed study of both the upper‐ and lower‐temperature nematic phases by using small‐angle X‐ray scattering. We observed a continuous change in *d* spacing rather than a sharp change at the phase transition, a result consistent with a transition between two nematic phases, structures of which are presumably degenerate.

## Introduction

The nematic liquid‐crystalline state—as was exhibited by low‐molar‐mass liquid crystals—is characterised by relatively high fluidity, a lack of positional ordering of molecules, but with short‐range orientational order. Transitions from one nematic phase into another are rare, but also highly topical due, in part, to the recent discovery of the twist‐bend nematic phase.^1^, ^2^, ^3^, ^4^, ^5^, ^6^ Several other nematic or nematic‐like mesophases are known to exist (chiral nematic (N*), discotic nematic (N_D_),^7^ re‐entrant nematic (N_RE_),^8^ biaxial nematic (N_B_),^9^, ^10^ blue phases (BPI, BPII, BPIII)) or are either predicted to exist or have possibly been discovered (cubatic nematic (N_cub_),^11^ splay‐bend nematic (N_SB_)^12^, ^13^). Recently, we have reported a polar liquid‐crystalline material (**1**, Table 1) that exhibited two distinct nematic mesophases, with a weak first‐order transition between the two phases.^14^ Other examples of nematic‐to‐nematic transitions have been observed in binary mixtures of re‐entrant materials,^15^ in frustrated chiral rod‐like systems^16^ and in main‐chain liquid crystal polymers.^17^ In terms of response to applied electric fields, the two nematic phases are similar, exhibiting a Fréedericksz transition with threshold voltages of approximately 0.3 V and approximately 0.45 V in the N and N_*X*_ phases, respectively. Connoscopy demonstrates the upper temperature nematic phase of **1** to be uniaxial and optically positive; however, following the N_*X*_–N transition, the homeotropic alignment is lost, and a schlieren texture obtained: therefore, it is unclear if this lower temperature nematic phase is uniaxial or biaxial at this time. For compound **1**, the N_*X*_–N transition occurs at 85.6 °C (determined by DSC); however, this is below the melting point of the material, and the mesophase is therefore metastable; if the properties and local structure of the N_*X*_ phase are to be understood, it is important that materials with superior working temperature ranges are developed.

**Table 1:** Transition temperatures, associated enthalpies of transition and dimensionless entropies of transition for compound **1**, as was determined using DSC at a heat/cool rate of 10 °C min^−1^.^14^ 
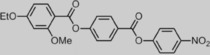

	MP	N_*X*_–N	*N*‐iso
*T* [°C]	139.0	85.6	182.1
Δ*H* [kJ mol^−1^]	34.8	0.2	0.6
Δ*S*/*R*	10.2	0.1	0.2

**Figure 1:**
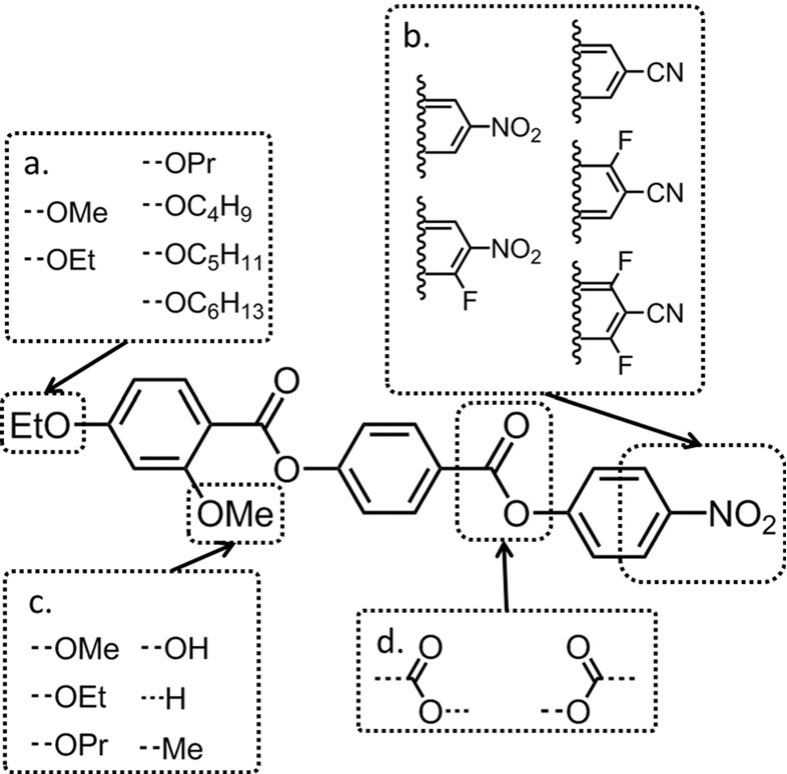
Proposed structural changes to **1**. a) Terminal chains. b) Polar terminal group(s). c) Lateral “bulky” group. d) Linking unit orientation.

Given that only one material is known to exhibit this nematic‐to‐nematic transition, a structure–property relationship is presently absent. Herein, we follow up on our earlier work by describing how the occurrence or absence of the N_*X*_ mesophase exhibited by compound **1** depends on molecular structure as shown in Figure 1, with the ultimate aim of preparing materials with superior working temperatures to that of the parent compound, and which could be subjected to in‐depth study across the N_*X*_–N phase transition.

## Experimental Section

4‐Hydroxy‐4′‐nitrobiphenyl was prepared as was described previously.^18^ Chemical reagents were purchased from commercial suppliers (Sigma Aldrich, TCI, Fluorochem and Apollo Scientific) and used without further purification. Solvents were purchased from Fisher Scientific and dried by percolation through activated alumina prior to use. Polarised optical microscopy was performed on a Zeiss Axioskop 40Pol microscope by using a Mettler FP82HT hotstage controlled by a Mettler FP90 central processor. Photomicrographs were captured via an InfinityX‐21 MP digital camera mounted atop the microscope. Differential scanning calorimetry (DSC) was performed on a Mettler DSC822^e^ calibrated before use against indium and zinc standards under an atmosphere of dry nitrogen, with DSC data then processed in Matlab. Computational chemistry was performed by using Gaussian G09 (Revision E.01)^19^ on the York Advanced Research Computing Cluster (YARCC), as was described in the text. Small‐angle X‐ray diffraction was performed by using a Bruker D8 Discover equipped with a temperature controlled, bored graphite rod furnace, custom built at the University of York. The radiation used was Cu_Kα_ (*λ*=0.154056 nm) from a 1 μS microfocus source. Diffraction patterns were recorded on a 2048×2048 pixel Bruker VANTEC 500 area detector set at a distance of 121 mm from the sample, allowing simultaneous collection of small angle and wide angle scattering data. Samples were filled into 0.9 mm O.D. capillary tubes and aligned with a pair of 1 T magnets, with the field direction being perpendicular to the incident X‐ray beam. Diffraction patterns were collected as a function of temperature, and the data was processed by using custom Matlab scripts. Raw data are available upon request from the University of York data catalogue. Full experimental details, including synthetic schemes and chemical characterisation, are given in the Supporting Information.

## Results and Discussion

Initially, we prepared a selection of compounds analogous in structure to **1** but with varying terminal chain lengths. Transition temperatures and phase identification were determined with a combination of polarised‐light optical microscopy (POM), differential scanning calorimetry (DSC) and variable temperature small angle X‐ray scattering (VT‐SAXS), as summarised below in Table 2.

**Table 2:** Transition temperatures [°C] and associated enthalpies of transition [kJ mol^−1^] for compounds **1**–**6**, as was determined by DSC at a heat/cool rate of 10 °C min^−1^. 
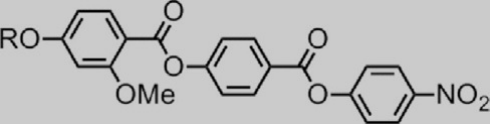

No.	R	Cr		N_*X*_		N		Iso
**1**	−C_2_H_5_	•	139.0 [34.5]	(•	85.6) [0.2]	•	182.1 [0.6]	•
**2**	−CH_3_	•	139.8 [29.9]	(•	132.7) [0.2]	•	187.9 [0.5]	•
**3**	−C_3_H_7_	•	134.7 [35.1]	(^[a]^	8.7±0.9)	•	161.2 [0.6]	•
**4**	−C_4_H_9_	•	111.4 [28.7]	–	–	•	160.3 [0.5]	•
**5**	−C_5_H_11_	•	98.8 [30.9]	–	–	•	150.3 [0.5]	•
**6**	−C_6_H_13_	•	109.6 [31.8]	–	–	•	148.5 [1.5]	•

**Figure 2:**
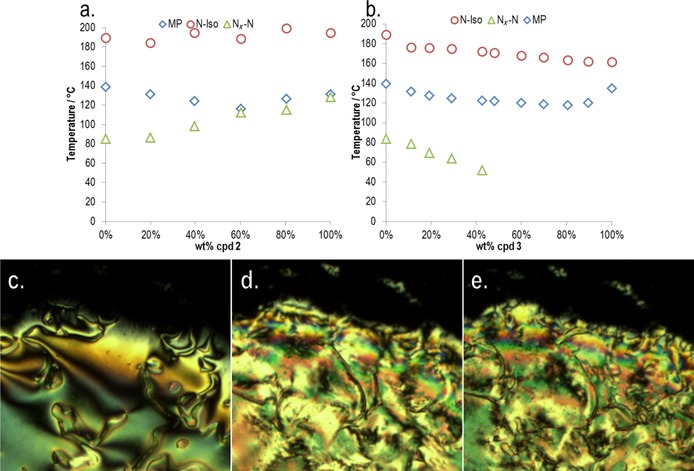
Gibbs phase diagram of binary mixtures (wt %) of **1** with a) **2** and b) c**3**. Linear fitting of the N_*X*_–N transition temperature as a function of concentration for the phase diagram between **1** and **3** gives a virtual transition temperature of 8.7 °C through the equation T_N*x*–N_=−75.9(±16.1) wt %+84.7(±4.0), *R* ^2^=0.987. Mixtures containing more than approximately 42 wt % of compound **3** crystallise prior to the onset of the N_*X*_ phase. Photomicrographs of **2** on untreated glass slides under crossed polarisers: in the nematic phase (c, 140 °C); at the N_*X*_–N transition (d, 132.6 °C); cooled deep into the N_*X*_ phase (e, 110 °C).

[a] An extrapolated “virtual” transition temperature was determined by linear fitting of T_N*x*_–N versus concentration (Figure 2 b); however, the material does not exhibit this phase in its neat state.

Increasing the length of the terminal chain from C2 in the parent compound led to the loss of the N_*X*_ phase along with modest reductions in melting point and clearing point, thereby indicating that the N_*X*_ phase is preferred when potential nanosegregation is minimised. Thus, shortening the terminal chain to C1 (i.e., OMe) affords compound **2**, and relative to the parent compound, this structural change gives a large increase in the onset temperature of the N_*X*_ phase. Representative photomicrographs are shown in Figure 2 c–e. The enthalpy associated with the N_*X*_–N transition for both **1** and **2** is vanishingly small (0.2 kJ mol^−1^ for both), and this results in the associated entropy of transition being extremely small for both materials (Δ*S*_N*x*–N_/R, 0.06 and 0.07 for **1** and **2**, respectively). As was noted by us previously, the small value of the enthalpy/entropy associated with the N_*X*_–N transition is consistent with a transition between two phases with the same macroscopic symmetry.^14^ A phase diagram was constructed between **1** and **2** as shown in Figure 2 a. Both compounds **1** and **2** were found to be miscible at all concentrations with both the *N*‐Iso and N_*X*_–N transition temperatures varying approximately linearly with concentration and therefore confirming the N_*X*_ phase is indeed exhibited by both materials. Given that a linear relationship exists between concentration and *T*_N*x*–N_, we were able to obtain “virtual” transition temperatures by extrapolation for materials that do not exhibit this mesophase or exhibit it at temperatures that are experimentally inaccessible. We also constructed a phase diagram between compounds **1** and **3** (Figure 2 b), both materials are miscible across all concentrations studied, but the N_*X*_ phase was found to decrease linearly with increasing concentration of **3**, disappearing at 43 wt %. By using a linear fit, we obtained a virtual (i.e., extrapolated) N_*X*_–N transition temperature of approximately 8.5 °C. As was demonstrated by the materials in Table 2, the N_*X*_–N transition apparently displays no odd‐even effect with regards to the terminal chain length, again confirming that nanosegregation associated with the aromatic to aliphatic proportions strongly influences mesophase formation.

We subjected compound **2** to analysis by SAXS with the intention of studying the change in scattering as a function of temperature across both nematic phases. Similar to compound **1**, we observed that the scattering of X‐rays by **2** was weak, and thus we required relatively long exposure times to obtain good signal‐to‐noise ratios. Despite the increase in the N_*X*_–N transition temperature afforded by compound **2**, we observed that the material was still prone to crystallisation during experimental studies, and so we were limited to collecting individual SAXS frames at specific temperatures rather than across the entire phase range.

In both the higher‐ and lower‐temperature nematic phases, the observed scattering was broadly similar to that reported previously for **1**;^14^ three diffuse peaks were seen at “small” angles parallel to the external aligning field (i.e., along the director, Figure 2 c). The positions of each of the three peaks were determined by deconvolution (Figure 4), the results are presented in Table 3. Although the *d* spacing of peak 1 (smallest value of *Q*) is comparable, if notably larger than, the molecular length of **2** (calculated to be 20.5 Å at DFT(B3LYP/6‐31G(d)) the other two peaks occur at significantly smaller *d* spacing.

**Table 3:** Peak positions (in *Q* [Å^−1^] and *d* [Å]) obtained by fitting scattered X‐ray intensity in parallel to the aligning magnetic field (i.e., the director) for **2** in the nematic and N_*X*_ phases at 140 and 129 °C, respectively.

Peak no.	N 140 °C [*Q*, Å^−1^]	N 140 °C [*d*, Å]	N_*X*_ 129 °C [*Q*, Å^−1^]	N_*X*_ 129 °C [*d*, Å]
1	0.2937	21.82	0.2978	21.09
2	0.6321	9.94	0.6620	10.10
3	0.9292	6.76	0.9244	6.80

**Figure 3:**
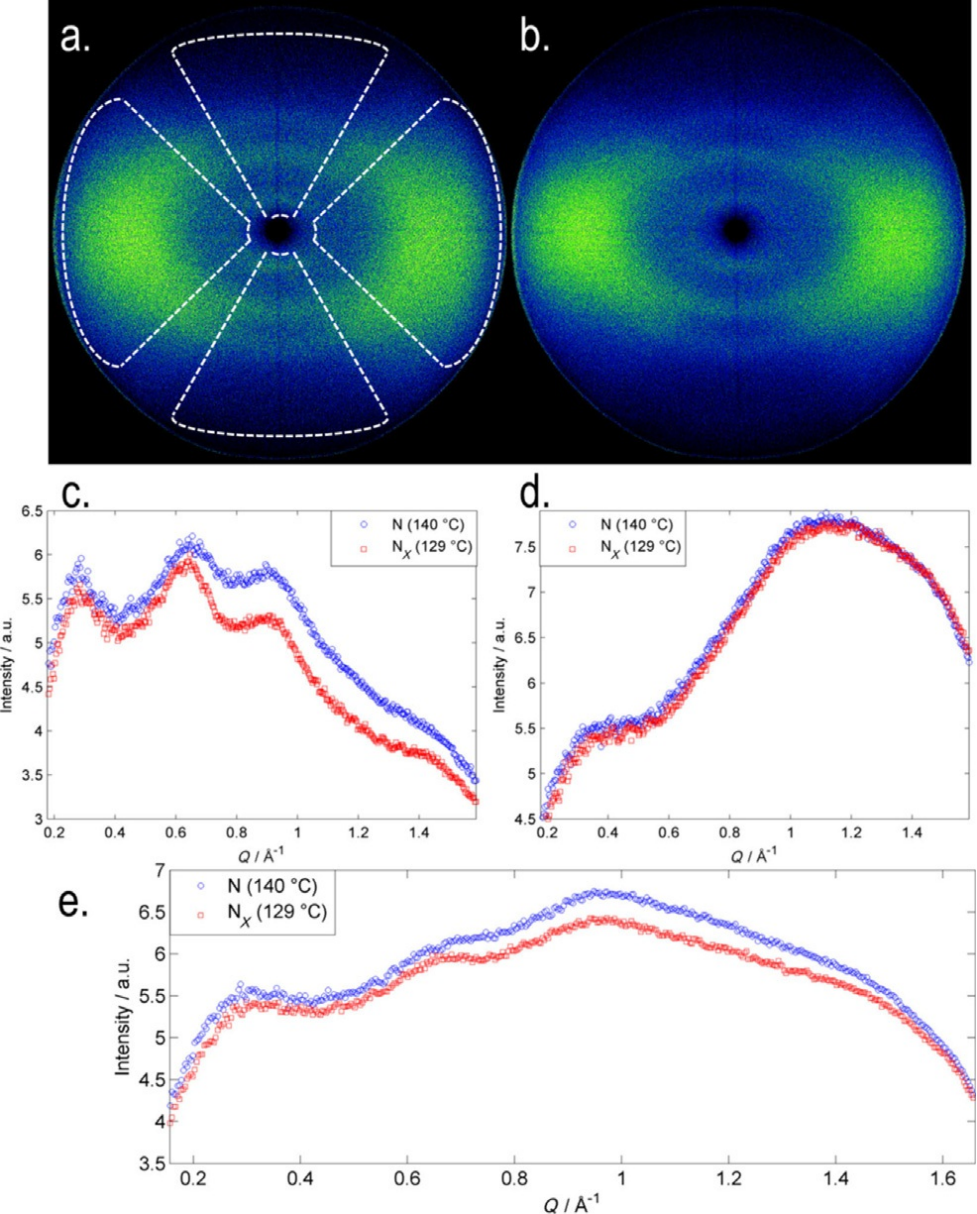
Analysis of compound **2** by small‐angle X‐ray scattering. a) Two dimensional scattering pattern obtained in the nematic phase at 140 °C. b) Two dimensional scattering pattern obtained in the N_*X*_ phase at 129 °C. c) Plot of scattered intensity parallel to the director as a function of *Q* (Å^−1^), obtained by radially averaging (0.05 ° steps) the upper and lower wedges illustrated on the nematic SAXS pattern. d) Plot of scattered intensity perpendicular to the director as a function of *Q* (Å^−1^), obtained by radially averaging (0.05 ° steps) the left and right wedges illustrated on the nematic SAXS pattern. e) Plot of scattered intensity obtained by radially averaging the entire SAXS pattern (0.05 °) steps showing how information is “lost” due to the overlapping of small‐ and wide‐angle scattering peaks.

**Figure 4:**
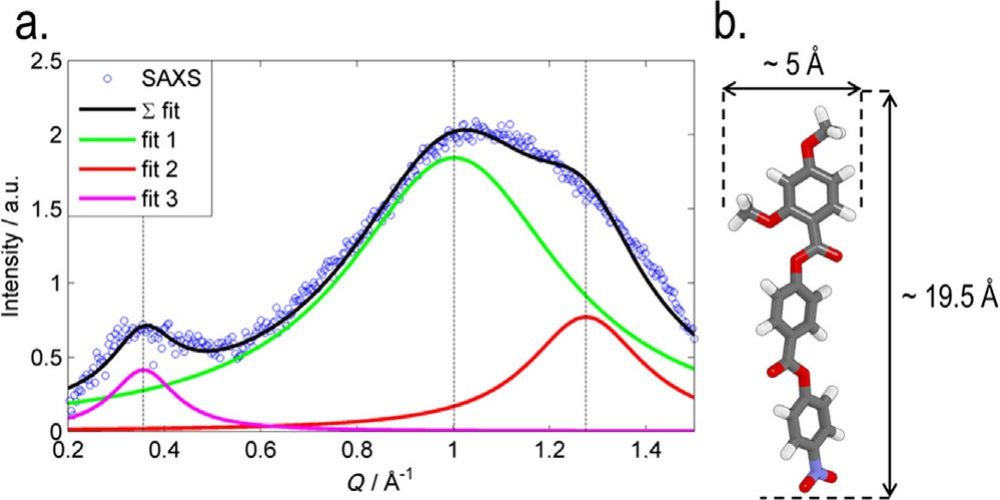
a) Fitting the scattered X‐ray intensity perpendicular to the aligning magnetic field for compound **2** in the nematic phase at 140 °C (*R* ^2^>0.99), b) the B3LYP/6‐31G(d) minimised geometry of **2** with the approximate molecular length and widths indicated.

In both the nematic and N_*X*_ phases, we observed two peaks at “wide” angles (i.e., perpendicular to the aligning field and the director, Figure 3 d); a broad peak at large values of *Q*, and a less intense broad peak at small values of *Q* (*Q*=0.3565 Å^−1^, *d*=17.7 Å). The scattering perpendicular to the aligning field is a consequence of the average lateral separation of the molecules, given that compound **1** has been demonstrated to form extensive antiparallel pairs, it is unsurprising that the wide‐angle peak is so broad, because many different forms of pairing are likely to exist (dimer, trimer, …*n*‐mer). The broad peak can be deconvoluted into two separate peaks (Figure 4, *Q*=1.0018 and 1.2755 Å^−1^, equal to *d*=6.3 and 4.9 Å, respectively); 4.9 Å is close to the width of an individual molecule and so we speculate that 6.3 Å is the width of a paired species. It is interesting to note that the position of each individual peak is the same in both the N and N_*X*_ phases; however, their relative size changes with the intensity of the peak at 6.3 Å increasing and that of the peak at 4.9 Å decreasing. If we assume that 4.9 and 6.3 Å are indeed the widths of an unpaired molecule and a paired species, respectively, then this suggests the degree of dimerisation is inversely proportional to temperature and therefore higher in the N_*X*_ phase than in the nematic, a result consistent with measurement of the Kirkwood factor of compound **1**.^14^

Next, we prepared a series of compounds with varying lateral group, allowing us to assess how variations to the steric bulk of the lateral unit impact upon the N_*X*_–N transition. We also studied how moving this lateral group from the 2‐ to the 3‐position of the left‐hand ring (**8**) impacts on mesomorphic behaviour. The melting properties for these compounds are given in Table 4 along with the parent material (**1**) for comparison.

**Table 4:** Transition temperatures [°C] and associated enthalpies of transition [kJ mol^−1^] for compounds **7**–**12**, in the case of compound **9** (denoted with a hash) the material begins to decompose before the clearing point at 240 °C is reached. An extrapolated “virtual” transition temperature was determined by linear fitting of T_N*x*_–N versus concentration for **7**, **9** and **10** (see Figure 6); however, these materials did not exhibit the N_*X*_ phase in their neat state. 
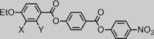

No.	X	Y	Cr		N_*X*_		N		Iso
**1**	H	OMe	•	139.0 [34.5]	•	85.6) [0.2]	•	182.1 [0.6]	•
**7**	H	H	•	155.2 [33.1]	(^[b]^	−17.4±1.0)	•	274.8 [0.6]	•
**8**	OMe	H	•	171.9 [48.7]	–	–	•	181.9 [0.3]	•
**9**	H	OH	•	164.7 [28.8]	(^[b]^	1.8±2.1)	•	>240^[a]^	•
**10**	H	CH_3_	•	138.8 [38.8]	(^[b]^	32.7±0.7)	•	186.2 [0.7]	•
**11**	H	OC_2_H_5_	•	143.3 [36.1]	(•	91.0 [0.2]	•	130.3) [0.6]	•
**12**	H	OC_3_H_7_	•	143.2 [40.6]	(•	77.6 [0.6]	•	99.2) [0.6]	•

[a] Transition temperatures [°C] and associated enthalpies of transition [kJ mol^−1^] for compounds **7**–**12**, in the case of compound **9** (denoted with a hash) the material begins to decompose before the clearing point at 240 °C is reached. [b] An extrapolated “virtual” transition temperature was determined by linear fitting of *T* ${}_{N{}_{X}-N}$ versus concentration for compounds **7**, **9** and **10** (Figure 6); however, these materials did not exhibit the N_*X*_ phase in their neat state.

**Figure 5:**
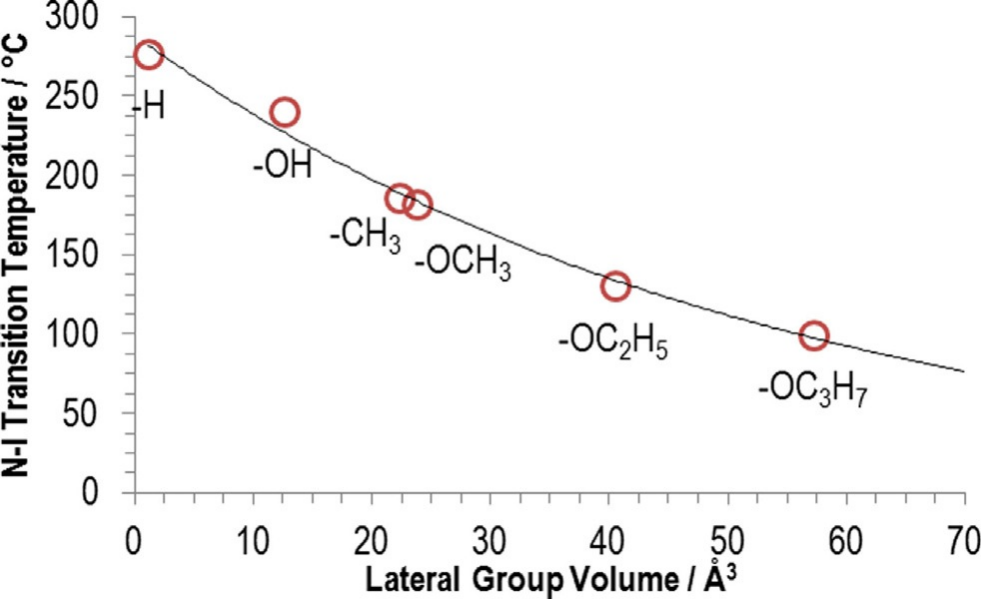
Plot of the *N*‐I transition temperature versus the volume of the lateral group (values from ref. 20) with an exponential fit (*Y*=288.37^−0.019 X^, *R* ^2^>0.99) to guide the eye.

Materials, in which the lateral group is smaller than methoxy (**7**, **9** and **10**) do not exhibit the N_*X*_ phase, whereas repositioning the methoxy group from the 2‐position to the 3‐position (**8**) also leads to the loss of the N_*X*_ phase. Increasing the length of the lateral alkyl chain, and hence the bulk volume, leads to a reduction in clearing points (Figure 5) and either a small increase (**11**) or decrease (**12**) in the temperature at which the N_*X*_–N transition occurs. Conversely, melting points do not appear to exhibit a dependence on the size of the lateral bulky group.

**Figure 6:**
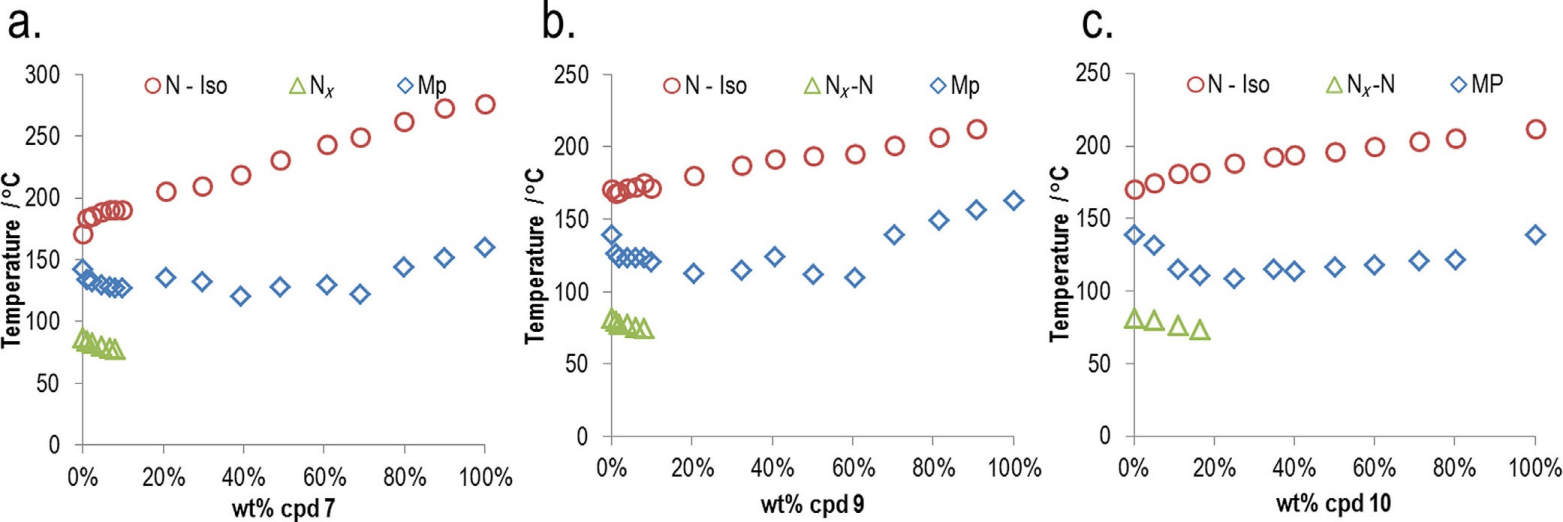
Gibbs phase diagram of binary mixtures (wt %) of compound **1** with a) **7**; b) **9**; and c) **10**. Linear fitting of the N_*X*_–N transition temperature as a function of concentration for each phase diagram gives a virtual N_*X*_–N transition temperatures of −23.8, 1.8 and 32.7 °C for **7**, **9** and **10** respectively. Equations of fit for **7** *T*_N*x*–N_=−102.57+85.168, *R* ^2^>0.99, for **9**: *T*_N*x*–N_=−78.611*x*+80.436, *R* ^2^>0.90, and for **10**: *T*_N*x*–N_=−50.442*x*+82.088, *R* ^2^>0.99. Mixtures containing more than approximately 10 wt % of **7**, approximately 10 wt % of **9** or approximately 20 wt % of **10** recrystallize prior to the N_*X*_–N phase transition.

Compounds **7**, **9** and **10** do not exhibit the N–N_*X*_ transition in their neat state, and so virtual transition temperatures were obtained by constructing phase diagrams between these materials and the standard compound **1**. Linear fitting of *T*_N*x*–N_ as a function of concentration affords the virtual transition temperature. Phase diagrams are presented in Figure 6, the virtual *T*_N*x*–N_ values were found to be, respectively, −23.8, 1.8 and 32.7 °C for **7**, **9** and **10**.

**Figure 7:**
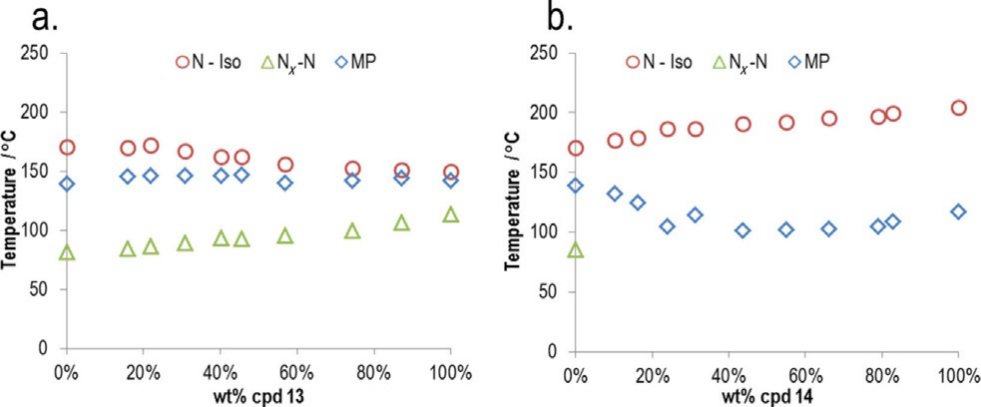
Gibbs phase diagram of binary mixtures (wt %) of **1** with a) **13** and b) **14**. For binary mixtures of **1**/**13** a) N_*X*_–N transition temperature varies approximately linearly with concentration, however, for mixtures of **1**/**14** b) addition of just 10 wt % of **14** suppresses the N_*X*_–N transition entirely.

We next explored how the magnitude of the lateral dipole moment influenced the N_*X*_–N transition by preparing compounds **13** and **14** (Table 5). Positioning a fluorine atom *ortho* to the nitro group in compound **1** gave **13**, which compared to the parent material exhibited a significant increase in the N_*X*_–N transition temperature, as well as a significantly reduced clearing point. Conversely, if a terminal nitrile is used in place of the nitro group, the N_*X*_ phase is suppressed. In an attempt to rationalise this, we calculated dipole moments at the B3LYP/6‐31G(d) level of DFT. The dipole moment of **13** is, predictably, larger (12.5 Debye) than that of both **1** (11.7 Debye) and **14** (11.5 Debye), suggesting that the magnitude of the dipole moment is important to the formation of this phase. Phase diagrams were constructed for binary mixtures of compound **1** with **13** and **14** (Figure 7). Across both phase diagrams, the clearing point was observed to vary linearly with concentration. For mixtures of **1** and **13**, the N_*X*_–N transition was found to vary linearly with concentration (*T*_NX–N_=30.393 *x*+80.043, *R* ^2^>0.96), indicating that the lower temperature mesophase exhibited by these two materials is the same. However, for mixtures of **1** and **14**, the N_*X*_ phase was not observed even at low concentrations of **14** (≈10 wt %).

**Table 5:** Transition temperatures [°C] and associated enthalpies of transition [kJ mol^−1^] for **13** and **14**. Phase transitions in parenthesis are monotropic, that is, they occur below the melting point of the sample. 
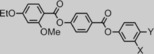

No.	X	Y	Cr		N_*X*_		N		Iso
**1**	H	NO_2_	•	139.0 [34.5]	(•	85.6) [0.2]	•	182.1 [0.6]	•
**13**	F	NO_2_	•	141.8 [43.7]	(•	117.1) [0.4]	•	149.6 [0.3]	•
**14**	H	CN	•	117.1 [41.2]	–	–	•	204.2 [1.1]	•

Studying compounds **1**, **13** and **14**, it is tempting to hypothesise about the role of the electric dipole moment: **13** has the largest dipole moment and the highest N_*X*_–N transition temperature; **1** has a smaller electric dipole moment than **13** and a lower N_*X*_–N transition temperature; **14** has a smaller dipole moment than **1** and does not exhibit the N_*X*_ phase. Although a small sample size, this hints that reducing the magnitude of the molecular electric dipole moment also serves to reduce the thermal stability of the N_*X*_ phase. As a test to this hypothesis, we prepared two materials, in one of which a carboxylate ester was removed (**15**), and another—in which a single carboxylate ester had its orientation “reversed” relative to that of the parent compound (**16**). Both structural modifications would be expected to reduce the molecular dipole moment, and we confirmed this with DFT(B3LYP/6‐31G(d)) calculated dipole moments. In the case of the modulated twist‐bend phase, this reversal of carboxylate esters was found to impact on the thermal stability of the TB phase.^21^ As will be discussed shortly, we also prepared several materials with larger dipole moments to test this hypothesis.

**Figure 8:**
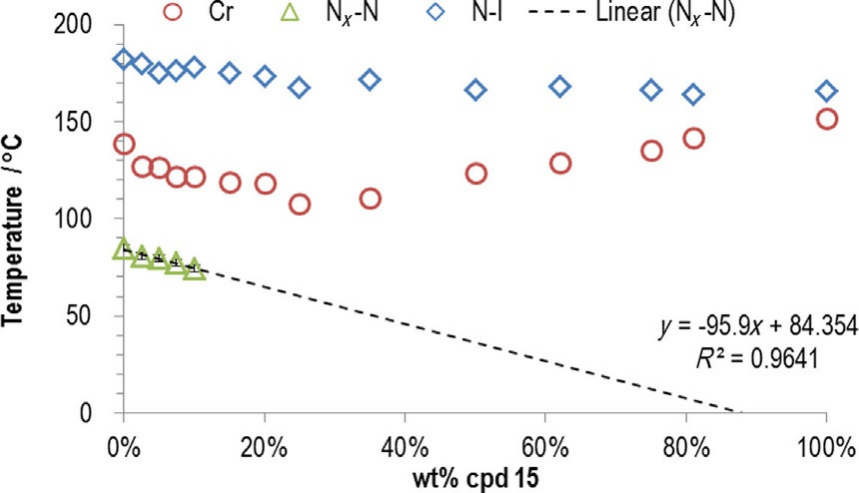
Gibbs phase diagram of binary mixtures (wt %) of **1** with **15**. Linear fitting of the N_*X*_–N transition temperature as a function of concentration (by the equation *T*_N*x*–N_=−95.9*x*+84.354, *R* ^2^>0.96) gives the virtual transition temperature of −11.6 °C. Mixtures containing more than approximately 10 wt % of **15** recrystallize prior to the N_*X*_–N phase transition; the error in this extrapolated value is therefore higher than for others in this work and is estimated to be ±7 °C.

With regards to the ester unit, its removal (to give the biphenyl benzoate **15**) or reversal (to give the phenyl 4‐nitrobenzoate **16**) results in the loss of the N_*X*_–N phase transformation and gave materials that are only nematogenic, with both **15** and **16** having a higher melting point than the parent **1**. Dipole moments were calculated at the B3LYP/6‐31G(dp) level to be 10.04 and 8.26 Debye for **15** and **16**, respectively. Both materials exhibit a nematic phase; however, neither exhibits the N_*X*_–N transition. Construction of a phase diagram between **1** and **15** and linear fitting of T_N*x*–N_ versus concentration gave the extrapolated value shown in Table 6, with the phase diagram presented in Figure 8.

**Table 6:** Transition temperatures [°C] and associated enthalpies of transition [kJ mol^−1^] for **15** and **16**. 
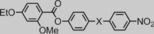

No.	X	Cr		N_*X*_		N		Iso
**1**	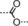	•	139.0 [34.5]	(•	85.6) [0.2]	•	182.1 [0.6]	•
**15**	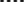	•	151.6 [40.9]	(^[a]^	−11.6±7.0)	•	165.9 [0.24]	•
**16**	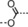	•	180.8 [45.8]	–	–	•	190.1 [0.40]	•

[a] An extrapolated “virtual” transition temperature was determined by linear fitting of T_N*x*_–N versus concentration for compound **15** (Figure 6); however, this material does not exhibit the N_*X*_ phase in its neat state.

Most structural modifications to the molecular structure of compound **1** were found to suppress the formation of the N_*X*_ phase, with the exception of reducing the length of the terminal ethoxy chain to methoxy (**2**) and increasing the dipole moment by incorporating a fluoro substituent *ortho* to the terminal nitro (**13**). Combining these two features gave **17**. Compounds **18**, **19** and **20** were prepared to further study how the molecular dipole moment and terminal groups influences the nematic and N_*X*_ phases. Transition temperatures and associated enthalpies of transition are given in Table 7.

**Table 7:** Transition temperatures [°C] and associated enthalpies of transition [kJ mol^−1^] for **17**–**21**. 
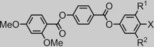

No.	X=	R^1^	R^2^	Cr		N_*X*_		N		Iso
**2**	NO_2_	H	H	•	139.8 [29.9]	(•	132.7) [0.2]	•	187.9 [0.5]	•
**17**	NO_2_	H	F	•	165.2 [50.1]	(•	139.6 [1.6]	•	155.2) [0.5]	•
**18**	CN	H	H	•	173.2 [41.0]	–	–	•	200.4 [0.7]	•
**19**	CN	F	H	•	197.3 [64.7]	–	–	(•	169.4) [0.5]	•
**20**	CN	F	F	•	162.6 [41.4]	–	–	(•	153.4) [0.6]	•
**21**	SF_5_	H	H	•	172.0 [40.4]	–	–	(•	93.1) [0.2]	•

Compared to the parent material, compound **17** has only a modest increase in the onset temperature for the N_*X*_ to nematic phase transition, with significantly reduced isotropisation temperature and a higher melting point. As was expected, the cyano‐terminated material **18** did not exhibit the N_*X*_ phase (mirroring the behaviour of **14**). Compounds **19** and **20** were prepared to determine if the incidence of the N_*X*_ phase is specific to nitro‐terminated materials, or if it can be formed by compounds with other suitably polar groups. There are several reports of liquid‐crystalline materials incorporating the pentafluorosulfanyl (SF_5_) group;^18^, ^22^, ^23^, ^24^, ^25^, ^26^ given the large dipole moment, we considered that a material analogous to **1**/**17** but with this terminal group in lieu of the nitro group may yield the N_*X*_–N polymorphism; however, compound **21** exhibited only a monotropic nematic phase, but did not exhibit a N_*X*_–N transition. Because both materials exhibited only a nematic phase, it would appear at present that a nitro group is essential, but given the limited set of compounds known to exhibit this phase, it is perhaps too early to draw firm property–structure correlations. Dipole moments for each of the materials presented in Table 5 were calculated at the B3LYP/6‐31G(dp) level of DFT: 11.37 Debye for **2**; 12.14 Debye for **17**; 11.13 Debye for **18**; 12.00 Debye for **19**; 12.84 Debye for **20**; 10.53 Debye for **21**. For the nitrile‐terminated compound **18**, the calculated dipole moment is intermediate between the two nitro‐terminated materials **2** and **18,** both of which exhibited the lower temperature nematic phase. The present results suggest that it is not the magnitude of the dipole moment of an individual molecule that dictates the incidence of the lower temperature nematic phase, but rather some property inherent to the nitro group, for instance, the extent of the delocalization of the electrons, and hence polarizability, over a broader functional group than nitrile.

One of our objectives was to obtain materials exhibiting an enantiotropic N_*X*_ phase, rendering them amenable to further study; however, this was not met. The thermal stability of the N_*X*_–N transition is highest when the material features short terminal chains and a highly polar terminal nitro group; however, these conditions also (predictably) lead to high melting points. Using transition temperatures and enthalpy data obtained by DSC, we used the modification to the Schroder van Laar equation reported by Raynes to predict both the composition and transition temperatures of the eutectic blends of a number of possible binary mixtures.^27^ This method predicted that the eutectic mixture of **2** and **17** should exhibit an enantiotropic N_*X*_ transition (N_*X*_–N 134.5 °C, with a melting point of 126.0 °C).

**Figure 9:**
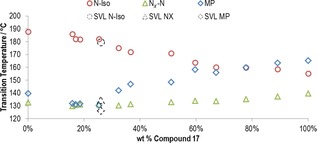
Gibbs phase diagram of binary mixtures (wt %) of **2** with **17**. The mixture with 25 wt % of **17** exhibited an enantiotropic N_*X*_–N transition over less than 0.5 °C. Predictions made by the SVL equation by using transitional data for **2** and **17** (obtained by DSC at a heat/cool rate of 10 °C min^−1^) predicts a eutectic mixture at 25.9 wt % of **17** in **2**; the predicted melting point (126.0 °C), N_*X*_–N transition temperature (134.5 °C) and clearing point (179.4 °C) are indicated in the phase diagram by dashed symbols.

The phase diagram of binary mixtures of **2**/**17** is shown in Figure 9. Although it was found that the experimental melting points were somewhat higher than predicted, the mixture containing approximately 24 wt % of **17** exhibited an enantiotropic N_*X*_ phase. Given the propensity of nitro‐terminated materials to form antiparallel pairs, it may be that considering the phase diagram as being “binary”, is misleading due to the formation of AA, AB and BB pairs in addition to the unpaired species (in which A/B=**2**/**17**), and this may be account for the underestimation of the melting point. Because the N_*X*_–N and *N*‐iso transition temperatures are simply a weighted average of the two pure components, we observed predicted values to be reasonably close to those determined experimentally.

**Figure 10:**
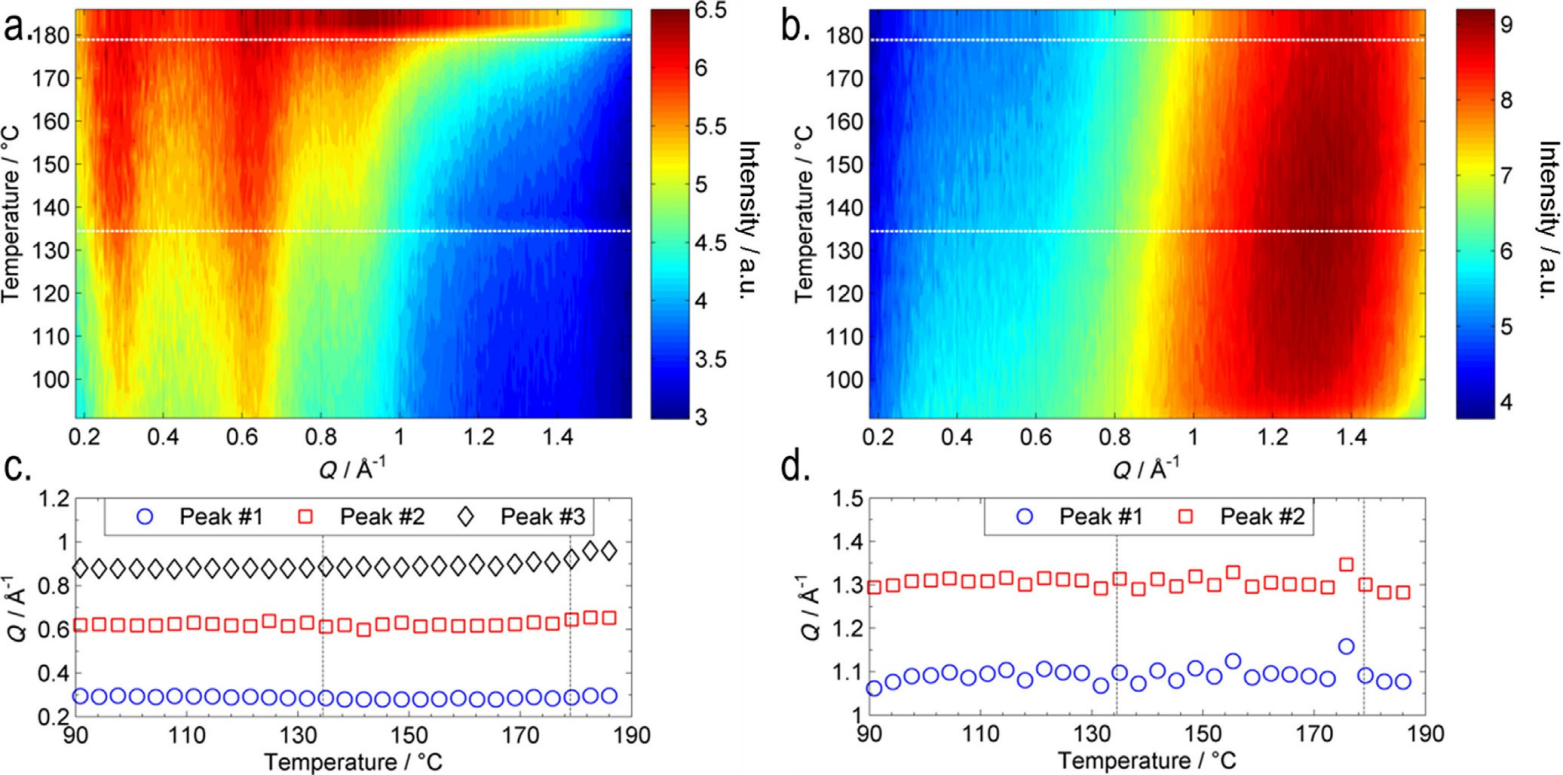
SAXS data for the eutectic mixture of **2**/**17**. a) Heatmap plot of scattered intensity parallel to the director as a function of temperature (°C) and *Q* (Å^−1^). b) Heatmap plot of scattered intensity perpendicular to the director as a function of temperature (°C) and *Q* (Å^−1^); c) plot of the *Q* (Å^−1^) value of the two major diffuse peaks in the small angle region as a function of temperature; d) plot of the *Q* (Å^−1^) value of the two major diffuse peaks in the wide‐angle region as a function of temperature. Dashed lines correspond to phase transitions.

Similar to initial study, we performed small angle X‐ray scattering as a function of temperature (3 °C steps), and by using the radial averaging procedure outlined in Figure 3, we separately obtained scattered intensity parallel and perpendicular to the director. Scattering data for the eutectic blend of **2**/**17** is presented as heatmap plots in Figure 10 for which the material was studied across a range of temperatures in 3 °C steps from the isotropic liquid (186 °C) deep into the N_*X*_ phase (91 °C). The temperatures at which phase transitions occurred are marked on the heatmap plots with dashed lines; upon cooling from the isotropic liquid into the nematic phase, there is a significant change in the scattering pattern; however, at the N_*X*_–N transition, there is little if any change. However, there is a continual reduction in the intensity of the small‐angle peaks as a function of temperature. The relatively weak scattering at small angles indicates that there is no build‐up of pretransitional cybotactic smectic domains within either the nematic or N_*X*_ phases.^28^, ^29^ Thus, the nematic‐to‐nematic transition described herein is presumably distinct from those observed in reentrant systems, such as those described in reference 15. Fitting the scattered intensity at each temperature allowed us to obtain the position of both small‐ and wide‐angle peaks. There is little change in peak positions at small angles (i.e., parallel to the director; Figure 10 c) upon cooling from the upper temperature nematic into the lower temperature “N_*X*_”. At wide angles (i.e., perpendicular to the director), the two peaks overlap significantly, and although the deconvoluted peaks do not shift in position (Figure 10 d), we observed that the intensity of peak #1 (*Q*≈1.1 Å^−1^, *d*≈5.7 Å) increased relative to that of peak #2 (*Q*≈1.3 Å^−1^, *d*≈4.8 Å), leading to a temperature‐dependent reduction in the *Q* value of the concurrent peak. Assuming that the earlier hypothesis that peak #2 is the width of a single molecule, and that peak #1 is the intermolecular spacing of a dimer pair then the this change in intensity can be understood as being a consequence of the increase in pairing of molecules as a function of temperature. This interpretation of the SAXS data is consistent with the measurement of the Kirkwood factor of compound **1**.

In our initial study on **1**, we noted that the N_*X*_–N transition could either occur as a consequence of a continual change in the concentration of dimers to unpaired molecules, or alternatively at the transition, there may be a discontinuous change in this concentration. The present SAXS data supports the former; the degree of pairing increases continually with reducing temperature, at some critical concentration the degree of pairing is sufficient to lead to the N_*X*_ phase, and a first‐order phase transition occurs. In this sense, the formation of the N_*X*_ phase proceeds via a comparable mechanism to that described by Cladis for the reentrant *n*OCB materials (i.e., 4‐alkoxy‐4′‐cyanobiphenyl, where *n* is the number of methylene units), although there is no intervening smectic mesophase in the present case. To date, we have only observed the N_*X*_ phase in nitro terminated materials, presumably some property of the nitro group leads to a specific pairing, which is crucial to the incidence of this phase.

## Conclusions

Compound **1** was previously demonstrated to exhibit two nematic mesophases (N_*X*_ and N) separated by a first‐order phase transition of small enthalpy; however, because the N_*X*_‐N transition is monotropic—occurring approximately 50 °C below the melting point—detailed study is complicated by crystallisation of the sample. Herein, we have investigated the molecular features that give rise to this phase sequence with a view to producing materials with superior working temperatures to compound **1**.

By using the onset temperature of the N_*X*_–N phase as a measure of the thermal stability of the lower temperature nematic, we found the following property–structure correlations: ) N_*X*_ mesophase is promoted by a short terminal chain (ethoxy or preferably methoxy); 2) terminal nitro group is essential; 3) thermal stability can be increased by positioning additional fluoro groups to enhance the molecular dipole moment; 4) use of other terminal polar groups (nitrile, pentafluorosulfanyl) or removal/reversal of carboxylate esters (which reduce the dipole moment) is detrimental to N_*X*_ phase formation; and v) lateral “bulky” group is required for a material to exhibit the N_*X*_ phase.

By using each of these correlations, we prepared a material designed to exhibit a high N_*X*_–N onset temperature (**17**), and although we observed significantly enhanced thermal stability of the N_*X*_ phase relative to that of parent compound (**1**), the material also exhibited a high melting point, which is perhaps unsurprising given the large dipole moment and short terminal chains. However, binary mixtures of **17** with **2** gave an eutectic mixture that exhibits an enantiotropic N_*X*_ phase. Studies of this material by X‐ray scattering confirmed the identity of both mesophases, and allowed us to present SAXS and WAXS data across the entire temperature range, and results suggests a continuous change in the degree of pairing rather than a jump at the N_*X*_–N phase transition.

## Conflict of interest

The authors declare no conflict of interest.

## Supporting information

As a service to our authors and readers, this journal provides supporting information supplied by the authors. Such materials are peer reviewed and may be re‐organized for online delivery, but are not copy‐edited or typeset. Technical support issues arising from supporting information (other than missing files) should be addressed to the authors.

Supplementary
