# Supplementary material for: Rational Design of Rod‐Like Liquid Crystals Exhibiting Two Nematic Phases
Source: Chemistry. 2017 Sep 18;23(58):14554–62. doi: 10.1002/chem.201702742 (PMC5656819; doi:10.1002/chem.201702742)
Supplement: Supplementary file 1 — Supplementary [file CHEM-23-14554-s001.pdf]

CHEMISTRY
A European Journal

Supporting Information

Rational Design of Rod-Like Liquid Crystals Exhibiting Two Nematic Phases

Richard J. Mandle,* Stephen J. Cowling, and John W. Goodby[a]

chem_201702742_sm_miscellaneous_information.pdf

1.1. General Methods

Chemical reagents were purchased from commercial suppliers and used without further purification. Solvents were purchased from Fisher Scientific UK and were dried *via* passage over activated alumina prior to use.

Reactions were monitored by thin layer chromatography (TLC) with DCM as the eluent. Silica coated aluminium TLC plates used were purchased from Merck (Kieselgel 60 F-254) and visualised using either UV light (254 nm and 365 nm), or by oxidation with either iodine or aqueous potassium permanganate solution. Yields refer to chromatographically (HPLC) and spectroscopically (^{1}H NMR, $^{13}C\{^{1}H\}$ NMR and where appropriate ^{19}F NMR) homogenous material. NMR spectra were recorded on a JEOL ECS spectrometer operating at 400 MHz (^{1}H), 100.5 MHz ($^{13}C\{^{1}H\}$ or 376.4 MHz (^{19}F)) as solutions in $CDCl_3$. Mass spectra were recorded on a Bruker micrOTOF MS-Agilent series 1200LC spectrometer. FTIR spectroscopy was performed using a Shimadzu IR Prestige-21 with Specac Golden Gate diamond ATR IR insert. High-performance liquid chromatography was performed on a Shimadzu Prominence modular HPLC system comprising a LC-20A solvent pump, a DGU-20A$_5$ degasser, a SIL-20A autosampler, a CBM-20A communication bus, a CTO-20A column oven, and a SPO-20A dual wavelength UV-vis detector operating at wavelengths of 230 and 255 nm. Reverse-phase HPLC was performed using an Alltech C18 bonded silica column with a 5 µm pore size, an internal diameter of 4.6 mm and a length of 250 mm, with neat acetonitrile used as the mobile phase. Chromatograms where only one peak was detected are quoted at >99% purity.

Polarised optical microscopy was performed on a Zeiss Axioskop 40Pol microscope using a Mettler FP82HT hotstage controlled by a Mettler FP90 central processor. Photomicrographs were captured *via* an InfinityX-21 MP digital camera mounted atop the microscope. Differential scanning calorimetry was performed on a Mettler DSC822^{e} calibrated before use against indium and zinc standards under an atmosphere of dry nitrogen. DSC thermograms were processed in Matlab. Computational chemistry was performed using the using Gaussian G09 revision d01 on the York Advanced Research Computing Cluster (YARCC) as described in the text. [2]

Small angle X-ray diffraction was performed using a Bruker D8 Discover equipped with a temperature controlled, bored graphite rod furnace, custom built at the University of York. The radiation used was copper Kα (λ = 0.154056 nm) from a 1 µS microfocus source. Diffraction patterns were recorded on a 2048x2048 pixel Bruker VANTEC 500 area detector set at a distance of 121 mm from the sample, allowing simultaneous collection of small angle and wide angle scattering data. Samples were filled into 1mm capillary tubes and aligned with a pair of 1T magnets. Diffraction patterns were collected as a

function of temperature and the data processed using Matlab. Raw data are available upon request from the University of York data catalogue.

1.1.1. General Esterification Method

The acid (1 eqv), phenol (1.5 eqv), EDAC.HCL or DCC (1.5 eqv) and DMAP (~ 0.1 eqv) were combined into a dried flask or reaction tube and DCM added. The suspension was vigorously stirred until the formation of the ester was complete as judged by TLC analysis (1 – 48 h). The reaction solution was concentrated, purified by flash chromatography and the chromatographed material recrystalised from an appropriate solvent.

1.1.2. General Williamson Etherification Method

The phenol (1 eqv), alkyl halide (> 1 eqv), potassium carbonate (2 eqv) and sodium iodide (1 eqv) were suspended in acetone and heated under reflux with vigorous stirring until complete consumption of the phenol as judged by TLC (24 - 48 h). The reaction was then cooled, diluted with DCM and filtered to remove insoluble matter. The crude material was then purified by flash chromatography (if required) and/or recrystalised from an appropriate solvent system.

1.1.3. General Ester Hydrolysis Method

The ester (1 eqv) and potassium hydroxide (> 2 eqv) were dissolved into ethanol/water (highest water concentration without precipitation) and heated under reflux until the complete consumption of the ester (TLC). The reaction was cooled, acidified with 2M HCl and the precipitate collected and air dried. If required the precipitate was recrystalised from an appropriate solvent system.

1.1.4. General Hydrogenation Method

The benzyl ester/ether (1 eqv) and palladium on carbon (10 wt %, 1 – 10 mol %) were placed into a flask evacuated with nitrogen gas. THF was added, the suspension degassed under vacuum with vigorous stirring and back filled with hydrogen (repeated three times). The reaction was left under an atmosphere of hydrogen for ~ 24h. The reaction was monitored by TLC analysis and once complete the catalyst was removed by filtering through a celite bed, the solvent removed and the crude material recrystalised from an appropriate solvent.

1.2. Characterisation of Liquid Crystalline Materials

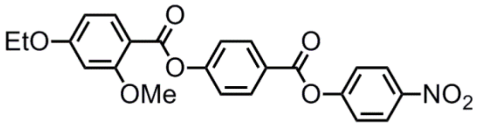

1: 4-((4-Nitrophenoxy)carbonyl)phenyl 4-ethoxy-2-methoxybenzoate

^{1}H NMR (400 MHz, $CDCl_3$): 1.40 (3H, t, J = 7.0, CH_3), 3.85 (3H, s, CH_3O), 4.06 (2H, quart, J = 7.0, CH_2O), 6.48 (1H, d, J = 2.1, Ar), 6.50 (1H, dd, J = 2.1, J = 8.9, Ar), 7.32 (2H, ddd, J = 2.1, J = 2.4, J = 8.9, Ar), 7.36 (2H, ddd, J = 2.1, J = 3.1, J = 9.2, Ar), 8.02 (1H, d, J = 8.9, Ar), 8.18 (2H, ddd, J = 2.1, J = 2.4, J = 2.9, Ar, 8.26 (2H, ddd, J = 2.1, J = 3.1, J = 9.2, Ar)

^{13}C{^{1}H} NMR (100.5 MHz, $CDCl_3$): 14.74, 56.11, 64.07, 99.51, 105.47, 110.18, 122.60, 122.74, 125.37, 125.61, 131.96, 134.71, 145.49, 155.77, 156.07, 162.62, 162.89, 163.71, 164.89

IR (ν_{max} cm^{-1}): 532, 617, 686, 748, 825, 1002, 1056, 1149, 1195, 1257, 1350, 1411, 1519, 1604, 1735, 2985, 3086, 3116

MS m/z (ESI+): 438.1185 (calcd. for $C_{23}H_{20}NO_8$: 438.1183, M+H),

Assay (HPLC): >99.9% (only one peak detected)

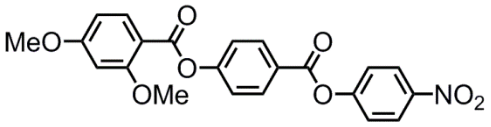

2: 4-((4-Nitrophenoxy)carbonyl)phenyl 2,4-dimethoxybenzoate

^{1}H NMR (400 MHz, $CDCl_3$):	3.89 (3H, s, OC<u>H</u>$_3$), 3.94 (3H, s, OC<u>H</u>$_3$), 6.54 (1H, d, *J* = 2.3 Hz, Ar<u>H</u>), 6.57 (1H, dd, *J* = 2.3 Hz, *J* = 8.5 Hz, Ar<u>H</u>), 7.38 (2H, ddd, *J* = 1.9 Hz, *J* = 2.5 Hz, *J* = 8.9 Hz, Ar<u>H</u>), 7.42 (2H, ddd, *J* = 2.2 Hz, *J* = 2.9 Hz, *J* = 9.2 Hz, Ar<u>H</u>), 8.10 (1H, d, *J* = 8.5 Hz, Ar<u>H</u>), 8.24 (2H, ddd, *J* = 1.9 Hz, *J* = 2.5 Hz, *J* = 8.9 Hz, Ar<u>H</u>), 8.33 (2H, ddd, *J* = 2.2 Hz, *J* = 2.9 Hz, *J* = 9.2 Hz, Ar<u>H</u>)
$^{13}C\{^{1}H\}$ NMR (100.5 MHz, $CDCl_3$):	55.78, 56.19, 99.12, 105.10, 110.44, 122.65,122.80, 125.43, 125.68, 132.02, 134.79, 145.54, 155.82, 156.10, 162.67, 162.92, 163.76, 165.52.
MS m/z (ESI+):	424.1030 (calcd. for $C_{21}H_{18}NO_8$: 424.1027, M+H)<br>446.0833 (calcd. for $C_{21}H_{17}NNaO_8$:446.0846, M + Na)
Assay (HPLC):	99.5%

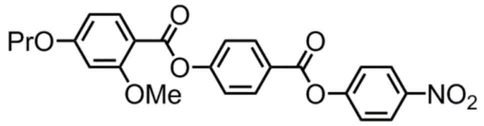

3: 4-((4-Nitrophenoxy)carbonyl)phenyl 4-propyloxy-2-methoxybenzoate (RM692)

^{1}H NMR (400 MHz, $CDCl_3$):	1.06 (3H, t, *J* = 7.4 Hz, $ArO\text{-}CH_2\text{-}CH_2\text{-}C\underline{H}_3$), 1.80 – 1.89 (2H, m, $ArO\text{-}CH_2\text{-}C\underline{H}_2\text{-}CH_3$), 3.92 (3H, S, $ArO\text{-}C\underline{H}_3$), 4.00 (2H, t, *J* = 7.4 Hz, $ArO\text{-}C\underline{H}_2\text{-}CH_2\text{-}CH_3$), 6.51 – 6.58 (2H, m, $Ar\underline{H}$), 7.37 (2H, ddd, *J* = 1.8 Hz, *J* = 2.5 Hz, *J* = 8.8 Hz, $Ar\underline{H}$), 7.42 (2H, ddd, *J* = 1.8 Hz, *J* = 3.6 Hz, *J* = 9.3 Hz, $Ar\underline{H}$), 8.07 (1H, d, *J* = 6.1 Hz, $Ar\underline{H}$)), 8.24 (2H, ddd, *J* = 1.8 Hz, *J* = 2.5 Hz, *J* = 8.8 Hz, $Ar\underline{H}$), 8.32 (2H, ddd, *J* = 1.8 Hz, *J* = 3.6 Hz, *J* = 9.3 Hz, $Ar\underline{H}$)
$^{13}C\{^1H\}$ NMR (100.5 MHz, $CDCl_3$):	10.58, 22.53, 22.54, 56.12, 70.00, 99.47, 105.51, 110.10, 122.62, 122.76, 125.37, 125.39, 125.40, 125.60, 131.96, 134.71, 145.50, 155.78, 156.09, 162.65, 162.89, 163.73, 165.11
MS m/z (ESI+):	452.131985 (calcd. for $C_{24}H_{22}NO_8$: 452.133993, M + H)<br>471.113122 (calcd. for $C_{24}H_{21}NNaO_8$: 474.115937, M + Na)
Assay (HPLC):	>99.9%

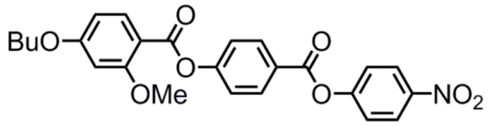

4: 4-((4-Nitrophenoxy)carbonyl)phenyl 4-butyloxy-2-methoxybenzoate

^{1}H NMR (400 MHz, $CDCl_3$):	0.93 (3H, t, *J* = 7.3, CH_3), 1.44 (2H, sext, *J* = 7.3, CH_2), 1.74 (2H, quint, *J* = 7.3, CH_2), 3.87 (3H, s, CH_3O), 3.99 (2H, t, *J* = 7.3, CH_2O), 6.47 (1H, d, *J* = 2.4, Ar), 6.50 (1H, dd, *J* = 2.4, *J* = 8.9, Ar), 7.32 (2H, ddd, *J* = 1.8, *J* = 2.8, *J* = 8.9, Ar), 7.36 (2H, ddd, *J* = 2.1, *J* = 3.1, *J* = 9.2, Ar), 8.02 (1H, d, *J* = 8.9, Ar), 8.19 (2H, ddd, *J* = 1.8, *J* = 2.8, *J* = 8.9, Ar), 8.27 (2H, ddd, *J* = 2.1, *J* = 3.1, *J* = 9.2, Ar)
^{13}C NMR (100.5 MHz, $CDCl_3$):	13.90, 19.27, 31.23, 56.11, 68.23, 99.50, 105.52, 110.14, 122.61, 122.74, 125.38, 125.61, 131.96, 134.69, 145.51, 150.73, 155.78, 156.09, 162.63, 162.89, 163.72, 165.11
IR (ν_{max} cm^{-1}):	686, 748, 833, 1002, 1126, 1195, 1342, 1465, 1512, 1597, 1735, 2862, 2939
MS m/z (ESI+):	488.1325 (calcd. for $C_{25}H_{23}NNaO_8$: 488.1316,M+Na)<br>466.1499 (calcd. for $C_{25}H_{24}NO_8$: 466.1496, M+H)
Assay (HPLC):	98.4%

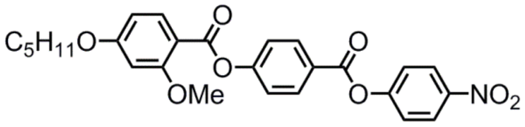

5: 4-((4-Nitrophenoxy)carbonyl)phenyl 4-pentyloxy-2-methoxybenzoate

^{1}H NMR (400 MHz, $CDCl_3$):	0.93 (3H, t, *J* = 7.0, CH_3), 1.33 – 1.50 (4H, m, CH_2CH_2), 1.81 (2H, quint, *J* = 7.0, CH_2), 3.91 (3H, s, CH_3O), 4.03 (2H, t, *J* = 7.0, CH_2O), 6.52 (1H, d, *J* = 2.1, Ar), 6.54 (1H, dd, *J* = 2.1, *J* = 8.9, Ar), 7.37 (2H, ddd, *J* = 1.8, *J* = 2.8, *J* = 8.5, Ar), 7.41 (2H, ddd, *J* = 1.8, *J* = 3.1, *J* = 9.2, Ar), 8.06 (1H, d, *J* = 8.9, Ar), 8.23 (2H, ddd, *J* = 1.8, *J* = 2.8, *J* = 8.5, Ar), 8.32 (2H, ddd, *J* = 1.8, *J* = 3.1, *J* = 9.2, Ar)
^{13}C NMR (100.5 MHz, $CDCl_3$):	14.08, 22.51, 28.21, 28.88 56.11, 68.23, 99.50, 105.52, 110.14, 122.60, 122.74, 125.37, 125.61, 131.95, 134.69, 145.51, 155.78, 156.00, 162.63, 162.89, 163.72, 165.11
IR (ν_{max} cm^{-1}):	648, 686, 748, 833, 864, 1002, 1056, 1134, 1195, 1257, 1342, 1465, 1504, 1566, 1597, 1735, 2322, 2630, 2769, 2854, 2931
MS m/z (ESI+):	502.1472 (calcd. for $C_{26}H_{25}NNaO_8$: 502.1472, M+Na), 480.1648 (calcd. for $C_{26}H_{26}NO_8$: 480.4930, M+H)
Assay (HPLC):	99.8%

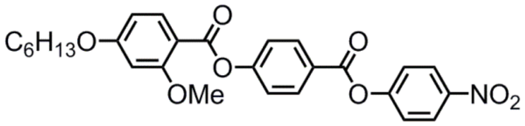

6: 4-((4-Nitrophenoxy)carbonyl)phenyl 4-hexyloxy-2-methoxybenzoate

^{1}H NMR (400 MHz, $CDCl_3$): 0.92 (3H, t, *J* = 7.0, CH_3), 1.32 – 1.41 (4H, m, CH_2CH_2), 1.48 (2H, quint, *J* = 7.0, CH_2), 1.81 (2H, quint, *J* = 7.0, CH_2), 3.93 (3H, s, CH_3O), 4.04 (2H, t, *J* = 7.0, CH_2O), 6.53 (1H, d, *J* = 2.1, Ar), 6.56 (1H, dd, *J* = 2.1, *J* = 8.5, Ar), 7.38 (2H, ddd, *J* = 2.1, *J* = 2.4, *J* = 8.9, Ar), 7.42 (2H, ddd, *J* = 2.1, *J* = 3.1, *J* = 9.2, Ar), 8.08 (1H, d, *J* = 8.5, Ar), 8.25 (2H, ddd, *J* = 2.1, *J* = 2.4, *J* = 8.9, Ar), 8.34 (2H, ddd, *J* = 2.1, *J* = 3.1, *J* = 9.2, Ar)

^{13}C NMR (100.5 MHz, $CDCl_3$): 14.10, 22.67, 25.74, 29.15, 31.62, 56.12, 68.55, 99.50, 105.53, 110.13, 122.60, 122.74, 125.37, 125.61, 131.95, 134.69, 145.51, 155.78, 156.09, 162.63, 162.89, 163.72, 165.11

IR (ν_{max} cm^{-1}): 501, 678, 748, 833, 1010, 1134, 1195, 1257, 1334, 1411, 1465, 1512, 1597, 1735, 2453, 2630, 2862, 2931, 3078

MS m/z (ESI+): 516.1638 (calcd. for $C_{27}H_{27}NNaO_8$: 516.1629, M+Na)
494.1816 (calcd. for $C_{27}H_{28}NO_8$: 494.1809, M+H)

Assay (HPLC): 99.3%

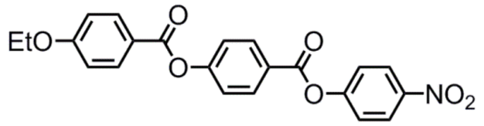

7: 4-((4-Nitrophenoxy)carbonyl)phenyl 4-ethoxybenzoate

^{1}H NMR (270 MHz, $CDCl_3$):	1.38 (3H, t, *J* = 7.0, CH_3), 4.08 (2H, quart, *J* = 7.0, CH_2O), 6.91 (2H, d, *J* = 8.5, Ar), 7.32 (2H, d, *J* = 8.5, Ar), 7.36 (2H, d, *J* = 8.9, Ar), 8.08 (2H, d, *J* = 8.5, Ar), 8.20 (2H, d, *J* = 8.5, Ar), 8.28 (2H, d, *J* = 8.9, Ar)
^{13}C NMR (67.5 MHz, $CDCll_3$):	14.62, 63.87, 114.40, 120.80, 122.62, 125.28, 131.96, 132.43, 154.42, 155.61, 155.84, 163.51, 163.66, 164.21
IR (v_{max} cm^{-1}):	501, 624, 686, 756, 848, 879, 879, 1010 1049, 1157, 1211, 1365, 1435, 1519.91, 1604, 1735, 2129.42, 2862, 2947, 3008, 3456
MS m/z (ESI+):	408.1078 (calcd. for $C_{22}H_{18}NO_7$: 408.3955, M+H),
Assay (HPLC):	99.4%

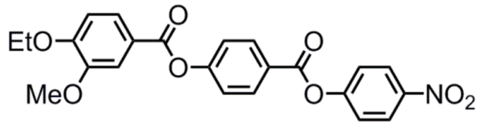

8: 4-((4-Nitrophenoxy)carbonyl)phenyl 4-ethoxy-3-methoxybenzoate

^{1}H NMR (400 MHz, $CDCl_3$): 1.30 (3H, t, *J* = 7.0, CH_3), 3.73 (3H, s, OCH_3), 3.98 (2H, q, *J* = 7.0, CH_2O), 6.76 (1H, d, *J* = 8.4, Ar), 7.11 – 7.28 (4H, m, Ar), 7.43 (1H, d, *J* = 1.5, Ar), 7.62 (1H, dd, *J* = 1.5, *J* = 8.4, Ar), 7.98 – 8.15 (4H, m, Ar)

^{13}C NMR (100.5 MHz, $CDCl_3$): 14.68, 56.23, 64.67, 111.44, 112.69, 120.95, 122.46, 122.71, 124.73, 125.39, 125.97, 132.09, 145.55, 149.13, 153.50, 155.72, 155.95, 163.61, 164.41

IR (ν_{max} cm^{-1}): 586, 686, 748, 810, 864, 1026, 1064, 1141, 1211, 1249, 1350, 1419, 1519, 1473, 1589, 1689, 1728, 2993, 3086

MS m/z (ESI+): 438.1139 (calcd. for $C_{23}H_{20}NO_8$: 438.1183, M+H)

Assay (HPLC): 99.7%

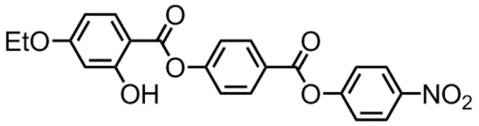

9: 4-((4-Nitrophenoxy)carbonyl)phenyl 2-hydroxy-4-ethoxybenzoate

^{1}H NMR (400 MHz,$CDCl_3$):	1.44 (3H, t, *J* = 7.0, CH_3), 4.07 (2H, quart, *J* = 7.0, CH_2), 6.46 (1H, d, *J* = 2.6, Ar), 6.50 (1H, dd, *J* = 2.6, *J* = 8.9, Ar), 7.36 (2H, d, *J* = 8.9, Ar), 7.40 (2H, dd, *J* = 9.3, Ar), 7.94 (1H, d, *J* = 8.9, Ar), 8.27 (2H, d, *J* = 8.9, Ar), 8.31 (2H, d, *J* = 9.3, Ar), 10.49 (1H, s, OH)
^{13}C NMR (67.5 MHz, $CDCl_3$):	14.53, 64.04, 101.27, 104.01, 108.75, 122.35, 122.61, 125.31, 126.30, 131.68, 132.03, 145.37, 154.91, 155.55, 163.39 164.69, 166.06, 167.96
IR (ν_{max} cm^{-1}):	501, 547, 640, 756, 840, 871, 933, 979, 1033, 1141, 1195, 1257, 1427, 1504, 1573, 1681, 2322, 2546, 2661, 2816, 2893, 2947, 3070, 3294
MS m/z (mTOF, ESI+):	424.1027 (calcd. for $C_{22}H_{18}NO_8$: 424.1027 M+H)
Assay (HPLC):	99.6%

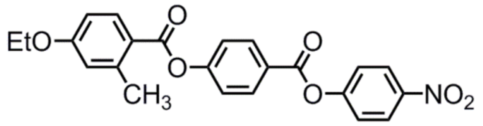

10: 4-((4-Nitrophenoxy)carbonyl)phenyl 4-ethoxy-2-methylbenzoate

^{1}H NMR (400 MHz, $CDCl_3$):	1.44 (3H, t, *J* = 7.0, CH_3), 2.64 (3H, s, Ar-CH_3), 4.10 (2H, q, *J* = 7.0, CH_2O), 6.78 – 6.83 (2H, m, Ar), 7.36 (2H, ddd, *J* = 1.8, *J* = 2.6, *J* = 8.8, Ar), 7.41 (2H, ddd, *J* = 2.2, *J* = 3.3, *J* = 9.2, Ar), 8.17 (1H, d, *J* = 9.5, Ar), 8.25 (2H, ddd, *J* = 1.8, *J* = 2.6, *J* = 8.8, Ar), 8.32 (2H, ddd, *J* = 2.2, *J* = 3.3, *J* = 9.2, Ar)
^{13}C NMR (100.5 MHz, $CDCl_3$):	14.77, 22.67, 63.80, 111.85, 117.88, 119.63, 122.59, 122.73, 125.39, 125.78, 132.05, 133.88, 144.89, 145.53, 155.74, 145.53, 155.74, 156.01, 162.82, 163.66, 164.50
IR (v_{max} cm^{-1}):	686, 756, 817, 879, 964, 1002, 1041, 1118 1157, 1203, 1265, 1350, 1419, 1519, 1604, 1712, 1928, 2985, 3086
MS m/z (ESI+):	422.1238 (calcd. for $C_{23}H_{20}NO_7$: 422.1234, M+H)
Assay (HPLC):	99.1%

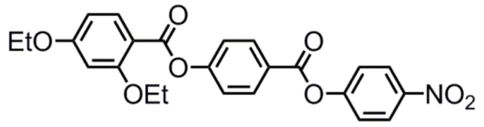

11: 4-((4-Nitrophenoxy)carbonyl)phenyl 2,4-diethoxybenzoate

^{1}H NMR (400 MHz, $CDCl_3$):	1.39 (3H, t, *J* = 7.0, CH_3), 1.43 (3H, t, *J* = 7.0, CH_3), 4.05 (2H, quart, *J* = 7.0, CH_2O), 4.07 (2H, quart, *J* = 7.0, CH_2O), 6.45 (1H, d, *J* = 2.1, Ar), 6.49 (1H, dd, *J* = 2.1, *J* = 8.9, Ar), 7.32 (2H, ddd, *J* = 2.1, *J* = 2.8, *J* = 8.9, Ar), 7.36 (2H, ddd, *J* = 2.1, *J* = 2.8, *J* = 2.9, Ar), 7.99 (1H, d, *J* = 8.9, Ar), 8.19 (2H, ddd, *J* = 2.1, *J* = 2.8, *J* = 8.9, Ar), 8.27 (2H, ddd, *J* = 2.1, *J* = 2.8, *J* = 2.9, Ar)
^{13}C NMR (100.5 MHz, $CDCl_3$):	14.65, 63.90, 64.58, 100.25, 105.40, 110.36, 122.49, 122.65, 125.28, 125.46, 145.39, 155.67, 156.05, 161.85, 163.02, 164.64
IR (ν_{max} cm^{-1}):	504, 563, 686, 748, 810, 879, 918, 1002, 1049, 1141, 1203, 1257, 1311, 1342, 1388, 1442, 1519, 1573, 1604, 1705, 1735, 2322, 2630, 2746, 2877, 2993, 3101, 3170, 3749
MS m/z (ESI+):	452.1337 (calcd. for $C_{24}H_{22}NO_8$: 452.1340, M+H)
Assay (HPLC):	99.9% (only one peak detected)

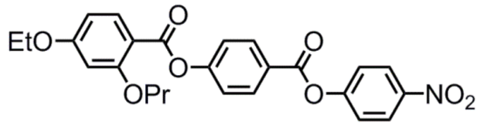

12: 4-((4-Nitrophenoxy)carbonyl)phenyl 4-ethoxy-2-propoxybenzoate

^{1}H NMR (400 MHz, $CDCl_3$): 0.99 (3H, t, *J* = 7.3, CH_3), 1.39 (3H, t, *J* = 7.0, CH_3), 1.81 (2H, sextet, *J* = 7.3, CH_2), 3.95 (2H, t, *J* = 7.3, CH_2O), 4.05 (2H, quart, *J* = 7.0, CH_2O), 6.44 (1H, d, *J* = 2.1, Ar), 6.47 (1H, dd, *J* = 2.1, *J* = 8.9, Ar), 7.31 (2H, ddd, *J* = 2.1, *J* = 2.4, *J* = 8.9, Ar), 7.35 (2H, ddd, *J* = 2.1, *J* = 3.0, *J* = 9.2, Ar), 7.98 (1H, d, *J* = 8.9, Ar), 8.19 (2H, ddd, *J* = 2.1, *J* = 2.4, *J* = 8.9, Ar), 8.26 (2H, ddd, *J* = 2.1, *J* = 3.0, *J* = 9.2, Ar)

^{13}C NMR (100.5 MHz, $CDCl_3$): 10.59, 14.65, 22.48, 63.90, 70.36, 100.09, 105.36, 110.34, 122.47, 122.64, 125.28, 125.46, 131.92, 134.61, 145.39, 155.67, 156.08, 161.93, 163.19, 163.62, 164.64

IR (ν_{max} cm^{-1}): 640, 686, 748, 802, 833, 887, 1002, 1056, 1149, 1195, 1257, 1504, 1604, 1728, 2877, 2939, 2970, 2993, 3078, 3109

MS m/z (mTOF, ESI+): 466.1497 (calcd. for $C_{25}H_{24}NO_8$: 466.1496, M+H)

Assay (HPLC): 99.9%

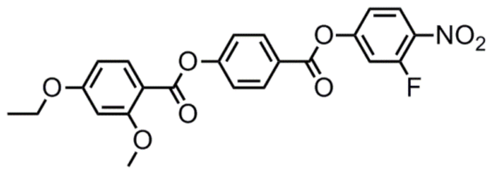

13: 4-((3-Fluoro-4-nitrophenoxy)carbonyl)phenyl 4-ethoxy-2-methoxybenzoate

^{1}H NMR (400 MHz, $CDCl_3$): 1.45 (3H, t, *J* = 7.0, CH_3), 3.91 (3H, s, OCH_3), 4.11 (2H, q, *J* = 7.0, CH_2O), 6.50 – 6.57 (2H, m, Ar), 7.21 (1H, m, Ar), 7.27 (1H, dd, *J* = 2.2, *J* = 11.2, Ar), 7.37 (2H, ddd, *J* = 2.2, *J* = 2.6, *J* = 8.4, Ar), 8.06 (1H, d, *J* = 8.8, Ar), 8.15 – 8.24 (3H, m , Ar)

^{13}C NMR (100.5 MHz, $CDCl_3$): 14.74, 56.11, 64.07, 99.53, 105.48, 110.15, 112.48 (d, *J* = 23.8, *C*-C-F), 118.18 (d, *J* = 3.8, *C*-C-C-F), 122.68, 125.16, 127.30 (d, *J* = 1.5, *C*-C-C-C-F), 132.02, 134.72, 134.92 (d, *J* = 7.7, *C*-C-C-F), 155.84 (d, *J* = 11.5, *O₂N-C*-C-F), 156.28, 156.30 (d, *J* = 267.0, *C*-F), 162.64, 162.83, 163.24, 164.92

^{19}F NMR (400 MHz, $CDCl_3$): -112.94 (dd, *J* =8.0, *J* = 10.3)

IR (v_{max} cm^{-1}): 686, 748, 810, 848, 1002, 1056, 1141, 1211, 1249, 1350, 1411, 1519, 1597, 1689, 1735, 2993, 3086

MS m/z (ESI+): 478.0914 (calcd. for $C_{23}H_{18}FNNaO_8$: 478.3836, M+Na)
456.1092 (calcd. for $C_{23}H_{19}FNO_8$: 456.1089, M+H)

Assay (HPLC) 99.3 %

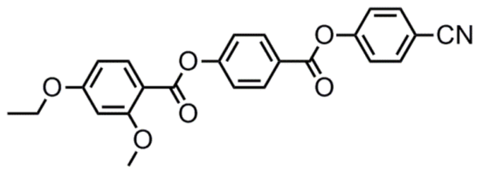

14: 4-((4-Cyanophenoxy)carbonyl)phenyl 4-ethoxy-2-methoxybenzoate

^{1}H NMR (400 MHz, $CDCl_3$): 1.45 (3H, t, J = 7.0, CH_3), 3.91 (3H, s, OCH_3), 4.11 (2H, q, J = 7.0, CH_2O), 6.52 (1H, d, J = 2.2, Ar) 6.54 (1H, dd, J = 2.2, J = 8.8, Ar) 7.35 (2H, ddd, J = 1.8, J = 2.6, J = 8.8, Ar), 8.06 (1H, d, J = 8.8, Ar), 8.22 (2H, ddd, J = 1.8, J = 2.6, J = 8.8, Ar)

^{13}C NMR (100.5 MHz, $CDCl_3$): 14.74, 56.11, 54.06, 99.53, 105.46, 109.93, 110.24, 118.36, 122.56, 123.03, 125.73, 131.92, 133.83, 134.71, 154.31, 156.60, 162.62, 162.88, 163.80, 164.88

IR (v_{max} cm^{-1}): 756, 871, 972, 1026, 1072, 1149, 1203, 1265, 1350, 1411, 1465, 1573, 1597, 1720, 2229, 2985, 3062

MS m/z (ESI+): 440.1112 (calcd. for $C_{24}H_{19}NNaO_4$:440.1105, M+Na)
418.1281 (calcd. for $C_{24}H_{20}NNaO_4$: 418.1285, M+H)

Assay (HPLC): 99.6%

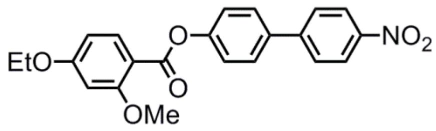

15: 4'-Nitro-[1,1'-biphenyl]-4-yl 4-ethoxy-2-methoxybenzoate

^{1}H NMR (400 MHz, $CDCl_3$):	1.44 (4H, t, *J* = 7.0, CH_3), 3.90 (3H, s, CH_3O), 4.10 (2H, quart, *J* = 7.0, CH_2O), 6.52 (1H, d, *J* = 2.4, Ar), 6.54 (1H, dd, *J* = 2.4, *J* = 8.5, Ar), 7.31 (2H, ddd, *J* = 2.1, *J* = 2.8, *J* = 8.9, Ar), 7.63 (2H, ddd, *J* = 2.1, *J* = 2.8, *J* = 8.9, Ar), 7.71 (2H, ddd, *J* = 2.1, *J* = 2.8, *J* = 8.9, Ar), 8.06 (1H, d, *J* = 8.5, Ar), 8.27 (2H, ddd, *J* = 2.1, *J* = 2.4, *J* = 8.9, Ar)
^{13}C NMR (100.5 MHz, $CDCl_3$):	14.68, 63.86, 64.59, 100.31, 105.35, 110.83, 122.80, 124.13, 127.71, 128.41, 134.44, 136.00, 146.95, 147.00, 151.90, 161.70, 163.71, 164.42
IR (v_{max} cm^{-1}):	532, 640, 686, 756, 810, 979, 1041, 1111, 1165, 1203, 1249, 1134, 1396, 1419, 1473, 1597, 1705, 2831, 2939, 2985, 3086
MS m/z (ESI+):	394.1291 (calcd. for $C_{22}H_{20}NO_6$: 394.1285, M+H)
Assay (HPLC):	99.9% (only one peak detected)

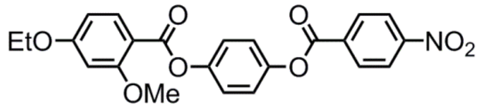

16: 4-((4-Nitrobenzoyl)oxy)phenyl 4-ethoxy-2-methoxybenzoate

^{1}H NMR (400 MHz, $CDCl_3$):	1.39 (3H, t, *J* = 7.0, CH_3), 3.85 (3H, S, OCH_3), 4.06 (2H, q, *J* = 7.0, CH_2O), 6.47 (1H, d, *J* = 2.2, Ar), 6.49 (1H, dd, *J* = 2.2, *J* = 8.4, Ar), 7.20 - 7.26 (4H, m, Ar), 8.00 (1H, d, *J* = 8.4, Ar), 8.26 - 8.38 (4H, m, Ar)
^{13}C NMR (100.5 MHz, $CDCl_3$):	14.75, 56.09, 64.00, 99.55, 105.34, 110.76, 112.23, 123.22, 123.82, 131.40, 134.59, 134.95, 147.68, 149.07, 151.00, 162.41, 163.33, 163.59, 164.61
IR (ν_{max} cm^{-1}):	648, 709, 756, 794, 840, 1010, 1072, 1141, 1165, 1203, 1242, 1265, 1342, 1465, 1504, 1527, 1566, 1604, 1728
MS m/z (ESI+):	460.0990 (calcd. for $C_{23}H_{19}NNaO_8$: 460.1003, M+Na)<br>438.1171 (calcd. for $C_{23}H_{20}NO_8$: 438.1183, M+H)
Assay (HPLC):	99.3%

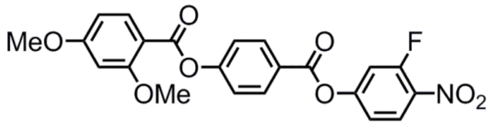

17: 4-((4-Nitro-3-fluorophenoxy)carbonyl)phenyl 2,4-dimethoxybenzoate

^{1}H NMR (400 MHz, $CDCl_3$):	3.91 (3H, s, ArOC<u>H</u>$_3$), 3.94 (3H, s, ArOC<u>H</u>$_3$), 6.55 (1H, d, *J* = 2.2 Hz, Ar<u>H</u>), 6.58 (1H, dd, *J* = 2.2 Hz, *J* = 8.5 Hz, Ar<u>H</u>), 7.23 (1H, ddd, *J* = 1.0 Hz, *J* = 2.3 Hz, *J* = 9.1 Hz, Ar<u>H</u>), 7.29 (1H, dd, *J* = 2.4 Hz, *J* = 11.2 Hz, Ar<u>H</u>), 7.39 (2H, ddd, *J* = 1.9 Hz, *J* = 2.3 Hz, *J* = 8.9 Hz, Ar<u>H</u>), 8.10 (1H, d, *J* = 8.5 Hz, Ar<u>H</u>), 8.16 – 8.26 (3H, m, Ar<u>H</u>)
^{13}C{^{1}H} NMR (100.5 MHz, $CDCl_3$):	55.42, 55.81, 98.72, 104.97, 109.99, 112.24 (d, *J* = 23.5 Hz), 118.10 (d, *J* = 3.5 Hz), 122.41, 124.98, 127.06, 131.72, 134.40, 134.64 (d, *J* = 8.8 Hz), 155.70, 155.93 (d, *J* = 266.5 Hz), 155.98, 162.35, 162.51, 162.94, 165.29
^{19}F NMR (376.4 MHz, $CDCl_3$):	-113.03 - -112.91 (m, Ar<u>F</u>)
MS m/z (ESI+):	442.0944 (calcd. for $C_{22}H_{17}FNO_8$: 442.0933, M + H)
	464.0748 (calcd. for $C_{22}H_{16}FNNaO_8$: 464.0752, M + Na)
Assay (HPLC):	99.4%

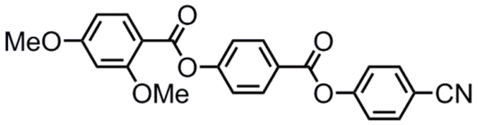

18: 4-((4-cyanophenoxy)carbonyl)phenyl 2,4-dimethoxybenzoate

^{1}H NMR (400 MHz, $CDCl_3$): 3.90 (3H, s, ArO-C<u>H</u>$_3$), 3.94 (3H, s, ArO-C<u>H</u>$_3$), 6.54 (1H, d, *J* = 2.1 Hz, Ar<u>H</u>), 6.58 (1H, dd, *J* = 2.1 Hz, *J* = 8.9 Hz, Ar<u>H</u>), 7.38 (2H, d, *J* = 8.5 Hz, Ar<u>H</u>), 7.75 (2H, d, *J* = 8.5 Hz, Ar<u>H</u>), 8.10 (1H, d, *J* = 8.9 Hz, Ar<u>H</u>), 8.25 (2H, d, *J* = 8.5 Hz, Ar<u>H</u>).

^{13}C{^{1}H} NMR (100.5 MHz, $CDCl_3$): 55.76, 56.17, 99.10, 105.09, 109.94, 110.43, 118.42, 122.60, 123.07, 125.77, 131.96, 133.87, 134.76, 154.32, 156.01, 162.63, 162.91, 163.83, 165.49.

MS m/z (ESI+): 404.1144 (calcd. for $C_{23}H_{18}NO_6$: 404.1129, M + H)
426.3801 (calcd. for $C_{23}H_{17}NNaO_6$: 426.3792, M + Na)

Assay (HPLC): 99.2%

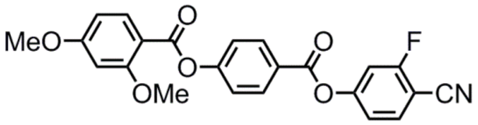

19: 4-((4-cyano-3-fluorophenoxy)carbonyl)phenyl 2,4-dimethoxybenzoate

^{1}H NMR (400 MHz, $CDCl_3$):	3.91 (3H, s, ArOC$\underline{H}_3$), 3.94 (3H, s, ArOC$\underline{H}_3$), 6.55 (1H, d, *J* = 2.2 Hz, Ar$\underline{H}$), 6.58 (1H, dd, *J* = 2.2 Hz, *J* = 8.7 Hz, Ar$\underline{H}$), 7.22 (2H, td, *J* = 2.0 Hz, *J* = 9.6 Hz, Ar$\underline{H}$), 7.37 (2H, ddd, *J* = 1.8 Hz, *J* = 2.5 Hz, *J* = 9.2 Hz, Ar$\underline{H}$), 7.68 – 7.74 (1H, m, Ar$\underline{H}$), 8.10 (1H, d, *J* = 9.6 Hz, Ar$\underline{H}$), 8.23 (2H, ddd, *J* = 1.8 Hz, *J* = 2.5 Hz, *J* = 9.2 Hz, Ar$\underline{H}$)
^{13}C{^{1}H} NMR (100.5 MHz, $CDCl_3$):	55.80, 56.20, 99.14, 105.11, 110.41, 111.25 (d, *J* = 22.7 Hz), 113.65, 118.93 (d, *J* = 3.7 Hz), 122.72, 125.35, 132.07, 134.26 (d, *J* = 1.0 Hz), 134.80, 156.24, 162.90, 163.39, 164.12 (d, *J* = 287.6 Hz)
^{19}F NMR (376.4 MHz, $CDCl_3$):	-103.16 (t, *J* = 8.1 Hz, Ar$\underline{F}$)
MS m/z (ESI+):	422.1051 (calcd for $C_{23}H_{17}FNO_6$: 422.1034, M + H)<br>444.0877 (calcd. for $C_{23}H_{16}FNNaO_6$:444.0854, M + Na)
Assay (HPLC):	99.6%

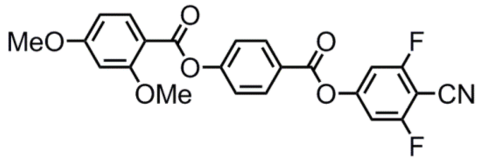

20: 4-((4-cyano-3,5-difluorophenoxy)carbonyl)phenyl 2,4-dimethoxybenzoate

^{1}H NMR (400 MHz, $CDCl_3$):	3.90 (3H, s, ArOC<u>H</u>$_3$), 3.94 (3H, s, ArOC<u>H</u>$_3$), 6.54 (1H, d, *J* = 2.2 Hz, Ar<u>H</u>), 6.58 (1H, dd, *J* = 2.2 Hz, *J* = 8.5 Hz, Ar<u>H</u>), 7.04 – 7.10 (2H, m, Ar<u>H</u>), 7.37 (2H, ddd, *J* = 1.8 Hz, *J* = 2.5 Hz, *J* = 8.5 Hz, Ar<u>H</u>), 7.68 – 7.74 (1H, m, Ar<u>H</u>), 8.10 (1H, d, *J* = 8.5 Hz, Ar<u>H</u>), 8.23 (2H, ddd, *J* = 1.8 Hz, *J* = 2.5 Hz, *J* = 8.5 Hz, Ar<u>H</u>)
^{13}C{^{1}H} NMR (100.5 MHz, $CDCl_3$):	55.77, 56.17, 99.10, 105.12, 107.11 (dd, *J* = 3.9 Hz, *J* = 23.0 Hz), 108.91, 110.28, 122.78, 124.87, 132.10, 134.77, 156.10 (t, *J* = 13.5 Hz), 156.41, 162.28, 162.85 (d, *J* = 10.4 Hz), 164.95 (dd, *J* = 6.6 Hz, *J* = 258.6 Hz), 165.56
^{19}F NMR (376.4 MHz, $CDCl_3$):	-101.87 (d, *J* = 8.4 Hz, Ar<u>F</u>)
MS m/z (ESI+):	440.0949 (calcd. for $C_{23}H_{16}F_2NO_6$: 440.0940, M + H)<br>462.0782 (calcd. for $C_{23}H_{15}F_2NNaO_6$: 462.0760, M + Na)
Assay (HPLC):	99.5%

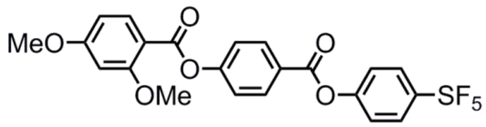

21: 4-((4-(pentafluoro-l6-sulfanyl)phenoxy)carbonyl)phenyl 2,4-dimethoxybenzoate

^{1}H NMR (400 MHz, $CDCl_3$):	3.80 (3H, s, $ArOC\underline{H}_3$), 3.84 (3H, s, $ArOC\underline{H}_3$), 6.45 (1H, d, *J* = 1.9 Hz, Ar$\underline{H}$), 6.48 (1H, dd, *J* = 1.9 Hz, *J* = 8.1 Hz, Ar$\underline{H}$), 7.21 – 7.30 (4H, m, Ar$\underline{H}$), 7.74 (2H, d, *J* = 8.5 Hz, Ar$\underline{H}$), 8.00 (1H, d, *J* = 8.1 Hz, Ar$\underline{H}$), 8.15 (2H, d, *J* = 8.5 Hz, Ar$\underline{H}$)
$^{13}C\{^{1}H\}$ NMR (100.5 MHz, $CDCl_3$):	55.74, 56.15, 99.10, 105.08, 110.46, 122.18, 122.57, 125.88, 127.72 (m), 131.95, 134.76, 150.94, 151.11, 151.29, 152.88, 155.96, 162.63, 162.93, 163.99, 165.48,
^{19}F NMR (376.4 MHz, $CDCl_3$):	63.63 (4F, dd, *J* = 17.2 Hz, *J* = 150.0 Hz, $S\underline{F_4}F$ (axial)), 84.97 (1F, quintet, *J* = 17.2 Hz, *J* = 150.0 Hz, $SF_4\underline{F}$ (equatorial)
MS m/z (ESI+):	505.0751 (calcd. for $C_{22}H_{18}F_5NO_6S$: 505.0739, M + H)<br>527.0572 (calcd. for $C_{23}H_{17}F_5NO_6S$: 527.0558, M + Na)
Assay (HPLC):	99.7%

1.3. Synthesis and Characterisation of Chemical Intermediates

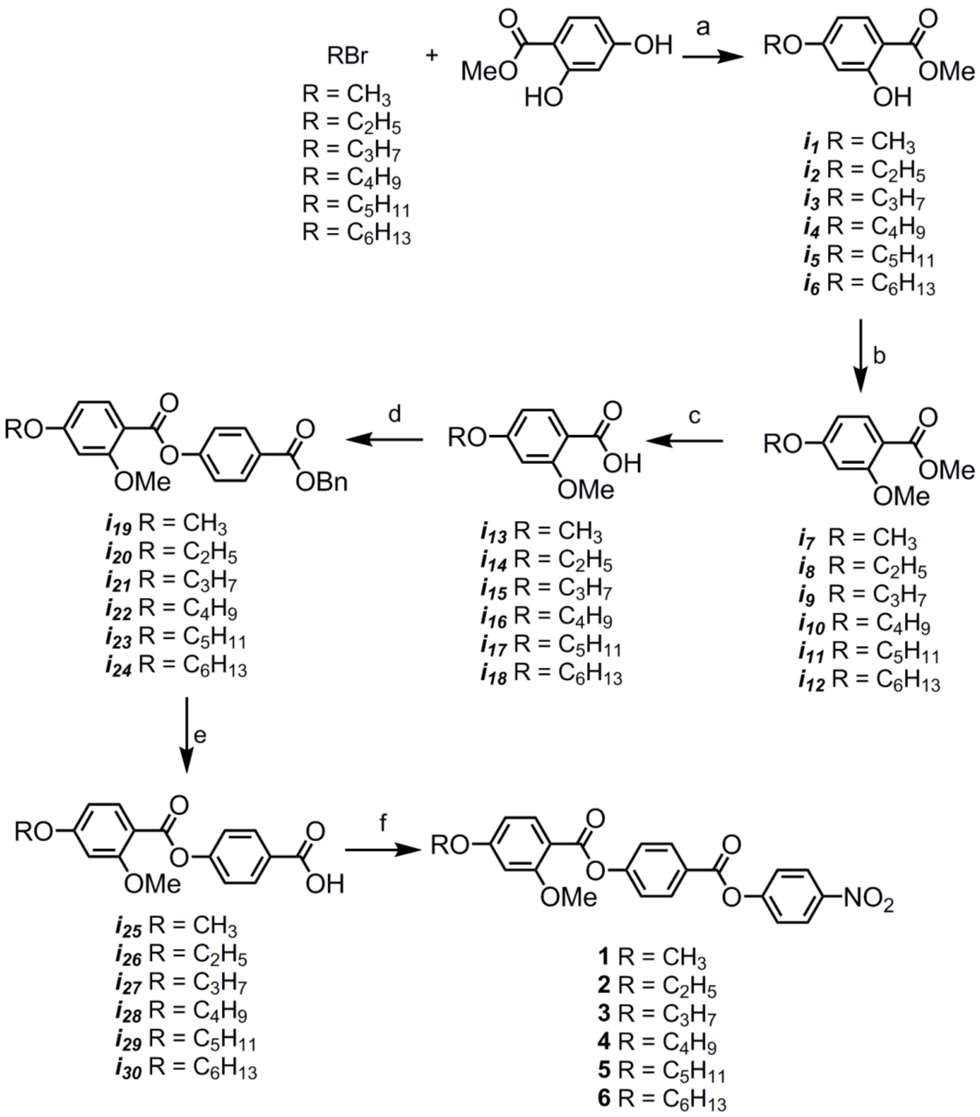

a...K_2CO_3, KI, Acetone
b... MeI, K_2CO_3, Acetone
c... (i)LiOH, H_2O, MeOH
(ii) HCl, H_2O
d... Benzyl 4-hydroxybenzoate, EDAC, DMAP, DCM
e... H_2, Pd/C, THF
f... 4-Nitrophenol, EDAC, DMAP, DCM

Scheme 1

i4: **Methyl 4-butyloxy-2-hydroxybenzoate**

Quantities used: 1-bromobutane (7.1 g, 5.6 ml, 52 mmol, methyl 2,4-dihydroxybenzoate (8.0 g, 0.52 mmol), potassium carbonate (14.3 g, 0.104 mol), potassium iodide (100 mg) and acetone (150 ml). The general Williamson etherification protocol was followed. Silica gel column chromatography with DCM as the eluent afforded compound ***i4*** as a pale yellow oil.

Yield: 8.2 g (70.4%)

^{1}H NMR (400 MHz, $CDCl_3$):	0.93 (3H, t, *J* = 7.3, CH_3), 1.46 (2H, sext, *J* = 7.3, CH_2), 1.73 (2H, quint, *J* = 7.3, CH_2), 3.86 (3H, s, $COOCH_3$), 3.93 (2H, q, *J* = 7.3, CH_2O), 6.37 (2H, m *J* = 2.4, *J* = 8.4, Ar), 7.67 (1H, d, *J* = 8.4, Ar), 10.93 (1H, s, OH)
^{13}C NMR (100.5 MHz, $CDCl_3$):	13.65, 19.05, 30.92, 51.75, 67.80, 100.97, 105.03, 107.73, 131.08, 163.65, 165.09, 170.29
IR (v_{max} cm^{-1}):	524, 640, 694, 748, 786, 840, 910, 956, 1002, 1095, 1134, 1180, 1249, 1334, 1435, 1504, 1573, 1612, 1674, 2607, 2870, 2939, 3163
MS m/z (mTOF, ESI+):	247.0941 ($C_{12}H_{16}NaO_4$, M+Na),<br>225.1126 ($C_{12}H_{17}O_4$, M+H)

i5: **Methyl 4-pentyloxy-2-hydroxybenzoate**

Quantities used: Compound 1-bromopentane (7.8 g, 6.5 ml, 52 mmol), methyl 2,4-dihydroxybenzoate (8.0 g, 0.52 mmol), potassium carbonate (14.3 g, 0.104 mol), potassium iodide (100 mg) and acetone (150 ml). The general Williamson etherification protocol was followed. Silica gel column chromatography with DCM as the eluent afforded compound ***i5*** as a pale yellow oil

Yield:	9.1 g (73.5%)
^{1}H NMR (400 MHz, $CDCl_3$):	0.90 (3H, t, *J* = 7.0, CH_3), 1.30 – 1.45 (4H, m, CH_2CH_2), 1.74 (2H, quint, *J* = 7.0, CH_2), 3.85 (3H, s, $COOCH_3$), 3.89 (2H, q, *J* = 7.0, CH_2O), 6.38 (2H, m, Ar), 7.67 (1H, m, Ar), 10.92 (1H, s, OH)
^{13}C NMR (100.5 MHz, $CDCl_3$):	13.84, 22.29, 27.97, 28.57, 51.72, 68.07, 100.94, 105.01, 107.70, 130.98, 163.64, 165.06, 170.27
IR (v_{max} cm^{-1}):	501, 540, 632, 694, 725, 779, 1141, 1219, 1350, 1442, 1504, 1581, 1620, 1666, 1743, 2322, 2870, 2954
MS m/z (mTOF, ESI+):	261.1097 ($C_{13}H_{18}NaO_4$, M+Na),<br>239.1281 ($C_{13}H_{19}O_4$, M+H)

i6: **Methyl 4-hexyloxy-2-hydroxybenzoate**

Quantities used: 1-bromohexane (8.6 g, 7.3 ml, 52 mmol), methyl 2,4-dihydroxybenzoate (8.0 g, 0.52 mmol), potassium carbonate (14.3 g, 0.104 mol), potassium iodide (100 mg) and acetone (150 ml). The general Williamson etherification protocol was followed. Silica gel column chromatography with DCM as the eluent afforded compound ***i6*** as a pale yellow oil

Yield: 10.2 g (77.8%)

^{1}H NMR (400 MHz, $CDCl_3$): 0.82 (3H, t, *J* = 6.4, CH_3), 1.21 – 1.29 (2H, m, CH_2), 1.31 – 1.40 (2H, m, CH_2), 1.68 (2H, quintet, *J* = 6.4, CH_2), 3.81 (3H, s, $COOCH_3$), 3.87 (2H, q, *J* = 6.4, CH_2O), 6.32 – 6.35 (2H, m, Ar), 7.63 (1H, d, *J* = 9.2, Ar), 10.88 (1H, s, OH)

^{13}C NMR (100.5 MHz, $CDCl_3$): 13.93, 22.51, 25.56, 27.89, 31.45, 51.82, 68.17, 101.01 105.08, 107.82, 131.05, 163.67, 165.13, 170.34

IR (v_{max} cm^{-1}): 594, 640, 694, 725, 779, 956, 1134, 1188, 1350, 1442, 1581, 1620, 1666, 1743, 2607, 2854, 2939, 3186

MS m/z (mTOF, ESI+): 275.1248 ($C_{14}H_{20}NaO_4$, M+Na),
253.1433 ($C_{14}H_{21}O_4$, M+H)

i10: **Methyl 2-methoxy-4-butyloxybenzoate**

Quantities used: Compound **i4** (4.75 g, 21.2mmol), methyl iodide (2.98 g, 1.31 ml, 21.2 mmol), potassium carbonate (5.85 g, 21.2 mmol), potassium iodide (100 mg), acetone (100 ml) and water (10 ml). The general Williamson etherification protocol was followed. Silica gel column chromatography with DCM as the eluent afforded compound ***i10*** as a pale yellow oil

Yield:	2.45 g (48.6%)
^{1}H NMR (400 MHz, $CDCl_3$):	0.90 (3H, t, *J* = 7.3, CH_3), 1.42 (2H, sext, *J* = 7.3, CH_2), 1.69 (2H, quint, *J* = 7.3, CH_2), 3.77 (3H, s, $COOCH_3$), 3.80 (3H, s, OCH_3), 3.91 (2H, q, *J* = 7.3, CH_2O), 6.37 – 6.42 (2H, m, Ar), 7.76 (1H, d, *J* = 9.5, Ar)
^{13}C NMR (100.5 MHz, $CDCl_3$):	13.57, 18.94, 30.92, 51.35, 55.65, 67.65, 99.03, 104.76, 111.65, 133.57, 161.09, 163.65, 165.83
IR (v_{max} cm^{-1}):	540, 632, 702, 771, 833, 987, 1033, 1087, 1141, 1249, 1435, 1573, 1604, 1720, 2870, 2954
MS (mTOF, ESI +):	261.1096 ($C_{13}H_{18}NaO_4$, M+Na)<br>239.1280 ($C_{13}H_{19}O_4$, M+H)

i11: **Methyl 2-methoxy-4-pentyloxybenzoate)**

Quantities used: ***i5*** (4.0 g, 16.8 mmol), methyl iodide (7.1 g, 3.1 ml, 50.4 mmol), potassium carbonate (4.6 g, 33.6 mmol), potassium iodide (100 mg), acetone (150 ml) and water (10 ml). The general Williamson etherification protocol was followed. Silica gel column chromatography with DCM as the eluent afforded compound ***i11*** as a pale yellow oil

Yield: 2.0 g (47%)

^{1}H NMR (400 MHz, $CDCl_3$): 0.90 (3H, t, *J* = 7.0, CH_3), 1.18 – 1.47 (4H, m, CH_2CH_2), 1.76 (2H, quint, *J* = 7.0, CH_2), 3.82 (3H, s, $COOCH_3$), 3.85 (3H, s, OCH_3), 3.96 (2H, q, *J* = 7.0, CH_2O), 6.42 – 6.47 (2H, m, *J* = 2.1, *J* = 9.2,Ar), 7.81 (1H, d, *J* = 9.2, Ar)

^{13}C NMR (100.5 MHz, $CDCl_3$): 13.94, 22.38, 28.08, 28.77, 51.61, 55.89, 68.17, 99.28, 104.93, 111.87, 133.78, 161.28, 163.83, 166.08

IR (ν_{max} cm^{-1}): 540, 632, 702, 771, 833, 964, 1033, 1087, 1141, 1249, 1435, 1465, 1573, 1604, 1720, 2870, 2947

MS (mTOF, ESI +): 275.1250 ($C_{14}H_{20}NaO_4$, M+Na),
253.1433 ($C_{14}H_{21}O_4$, M+H)

i12: **Methyl 2-methoxy-4-hexyloxybenzoate**

Quantities used: ***i6*** (4.0 g, 15.9 mmol), methyl iodide (6.7 g, 2.9 ml, 47.6 mmol), potassium carbonate (4.4 g, 31.7 mmol), potassium iodide (100 mg), acetone (150 ml) and water (10 ml). The general Williamson etherification protocol was followed. Silica gel column chromatography with DCM as the eluent afforded compound ***i12*** as a pale yellow oil

Yield:	3.8 g (90%)
^{1}H NMR (400 MHz, $CDCl_3$):	0.88 (3H, t, *J* = 6.7, CH_3), 1.21 – 1.33 (4H, m, CH_2CH_2), 1.39 (2H, quint, *J* = 6.7, CH_2), 1.71 (2H, quint, *J* = 6.7, CH_2), 3.77 (3H, s, $COOCH_3$), 3.86 (3H, s, OCH_3), 3.91 (2H, q, *J* = 6.7, CH_2O), 6.40 (2H, m, Ar), 7.77 (1H, m, Ar)
^{13}C NMR (100.5 MHz, $CDCl_3$):	14.00, 22.54, 25.63, 29.06, 31.51, 51.64, 55.92, 68.22, 99.31, 104.95, 111.89, 133.80, 161.29, 163.84, 166.10
IR (v_{max} cm^{-1}):	540, 632, 833, 1033, 1087, 1141, 1203, 1249, 1435, 1465, 1573, 1604, 1720, 2862, 2931
MS (m/z, ESI+):	289.1404 ($C_{15}H_{22}O_4$, M + Na)<br>267.1599 ($C_{15}H_{23}O_4$, M + H)

i16: **2-Methoxy-4-butyloxybenzoic acid**

Quantities used: ***i10*** (2.3 g, 9.66 mmol), potassium hydroxide (2.16 g, 38.65 mmol), methanol (100 ml) and water (10 ml). The general ester hydrolysis protocol was observed, affording the title compound as a white solid following recrystalisation from ethanol.

Yield:	2.1 g (89%)
Melting Point (°C):	95.7
^{1}H NMR (400 MHz, DMSO):	0.91 (3H, t, *J* = 7.3, CH_3), 1.41 (2H, sext, *J* = 7.3, CH_2), 1.70 (2H, quint, *J* = 7.3, CH_2), 3.80 (3H, s, CH_3O), 3.96 (2H, t, *J* = 7.3, CH_2O), 6.41 – 6.49 (2H, m, Ar), 7.70 (1H, d, *J* = 8.5, Ar)
^{13}C NMR (100.5 MHz, DMSO):	13.47, 18.59, 30.56, 55.51, 67.30, 98.81, 105.10, 111.94, 133.35, 160.49, 163.16, 166.23
IR (v_{max} cm^{-1}):	555, 609, 694, 748, 833, 902, 956, 1026, 1095, 1203, 1265, 1419, 1465, 1566, 1597, 1666, 2322, 2538, 2615, 2816, 2870, 2939
MS (m/z, ESI +),	247.0936 ($C_{12}H_{16}NaO_4$, M+Na),<br>225.1121 ($C_{12}H_{17}O_4$, M+H)

i17: **2-Methoxy-4-pentyloxybenzoic acid**

Quantities used: ***i11*** (2.0 g, 8.4 mmol), potassium hydroxide (1.9 g, 33.6 mmol), methanol (100 ml) and water (10 ml). The general ester hydrolysis protocol was observed, affording the title compound as a white solid following recrystalisation from ethanol.

Yield: 1.8 g (90%)

^{1}H NMR (400 MHz, DMSO): 0.83 (3H, t, *J* = 7.3, CH_3), 1.25 – 1.41 (4H, m, CH_2CH_2), 1.70 (2H, quint, *J* = 7.3, CH_2), 3.81 (3H, s, CH_3O), 3.92 (2H, t, *J* = 7.3, CH_2O), 6.40 – 6.46 (2H, m ,Ar), 7.73 (1H, d, *J* = 8.5, Ar)

^{13}C NMR (100.5 MHz, DMSO): 13.62, 21.80, 27.52, 28.19, 55.51, 67.61, 98.70, 105.60, 111.67, 133.46, 160.45, 163.25

IR (ν_{max} cm^{-1}): 501, 555, 640, 740, 810, 948, 1018, 1095, 1157, 1203, 1242, 1419, 1465, 1573, 1597, 1666, 1828, 2052, 2167, 2322, 2546, 2615, 2862, 2931

MS (mTOF ESI +), 239.1278 ($C_{13}H_{19}O_4$, M+H)

i18: **2-Methoxy-4-hexyloxybenzoic acid**

Quantities used: ***i12*** (3.8 g, 14.3 mmol), potassium hydroxide (3.2 g, 57.1 mmol), methanol (100 ml) and water (10 ml). The general ester hydrolysis protocol was observed, affording the title compound as a white solid following recrystalisation from ethanol.

Yield:	3.2 g (88%)
^{1}H NMR (400 MHz, DMSO):	0.87 (3H, t, *J* = 7.1, CH_3), 1.22 – 1.32 (4H, m, CH_2CH_2), 1.38 (2H, quint, *J* = 7.1, CH_2), 1.69 (2H, quint, *J* = 7.1, CH_2), 3.79 (3H, s, CH_3O), 3.95 (2H, t, *J* = 7.3, CH_2O), 6.45 (1H, dd, *J* = 2.1, *J* = 8.5, Ar), 6.48 (1H, d, *J* = 2.1 Ar), 7.70 (1H, d, *J* = 8.5, Ar)
^{13}C NMR (100.5 MHz, DMSO):	13.75, 22.04, 25.12, 28.54, 30.97, 55.55, 67.67, 98.58, 105.17, 112.05, 133.37, 160.55, 163.19, 166.28
IR (ν_{max} cm^{-1}):	532, 609, 756, 956, 1018, 1095, 1265, 1404, 1573, 1604, 1658, 2322, 22538, 2654, 2870, 2947
MS (mTOF ESI +),	253.1440 ($C_{14}H_{21}O_4$, M+H)

i22: **4-((Benzyloxy)carbonyl)phenyl 2-methoxy-4-butyloxybenzoate**

Quantities used: ***i16*** (1.0 g, 4.46 mmol), benzyl 4-hydroxybenzoate (1.5 g, 6.69 mmol), EDAC (1.3 g, 6.69 mmol), DMAP (6 mg, 50 µmol) and DCM (50 ml). The general Steglich esterification protocol was followed. Column chromatography over silica with 17:3 DCM/hexane as the eluent gave ***i22*** as a viscous oil.

Yield: 1.1 g (55%)

^{1}H NMR (400 MHz, $CDCl_3$): 0.92 (3H, t, *J* = 7.3, CH_3), 1.43 (2H, sext, *J* = 7.3, CH_2), 1.72 (2H, quint, *J* = 7.3, CH_2) 3.84 (3H, s, CH_3O), 3.96 (2H, quart, *J* = 7.3, CH_2O), 5.29 (2H, s, Bn), 6.44 (1H, d, *J* = 2.3,Ar), 6.46 (1H, dd, *J* = 2.3, *J* = 8.7, Ar), 7.20 (2H, ddd, *J* = 2.3, *J* = 2.9, *J* = 9.2, Ar), 7.24 - 7.42 (5H, m, Bn), 7.98 (1H, d, *J* = 8.7, Ar), 8.05 (2H, ddd, *J* = 2.3, *J* = 2.9, *J* = 9.2 Ar)

IR (ν_{max} cm^{-1}): 632, 694, 756, 833, 879, 1002, 1111, 1157, 1195, 1265, 1458, 1573, 1604, 1712, 2623, 2870, 2954

MS m/z (mTOF, ESI+): 435.1809 ($C_{26}H_{27}O_6$, M+H)

i23: **4-((Benzyloxy)carbonyl)phenyl 2-methoxy-4-pentyloxybenzoate**

Quantities used: ***i17*** (1.0 g, 4.2 mmol), benzyl 4-hydroxybenzoate (1.44 g, 6.3 mmol), EDAC (1.2 g, 6.3 mmol), DMAP (6 mg, 50 µmol) and DCM (50 ml). The general Steglich esterification protocol was followed. Column chromatography over silica with 17:3 DCM/hexane as the eluent gave ***i23*** as a viscous oil.

Yield: 0.7 g (37%)

^{1}H NMR (400 MHz, $CDCl_3$): 0.92 (3H, t, *J* = 7.0, CH_3), 1.33 – 1.49 (4H, m, CH_2CH_2), 1.80 (2H, quint, *J* = 7.0, CH_2) 3.89 (3H, s, CH_3O), 4.01 (2H, quart, *J* = 7.0, CH_2O), 5.35 (2H, s, Bn), 6.49 – 6.54 (2H {1H+1H}, m {d+dd}, *J* = 2.4, *J* = 2.4, *J* = 8.5,Ar), 7.26 (2H, ddd, *J* = 1.8, *J* = 2.4, *J* = 8.9, Ar), 7.30 - 7.46 (5H, m, Bn), 8.04 (1H, d, *J* = 8.5, Ar), 8.11 (2H, ddd, *J* = 1.8, *J* = 2.4, *J* = 8.9 Ar)

13C NMR (100.5 MHz, CDCl3): 14.10, 22.52, 28.21, 28.88, 56.09, 66.84, 68.49, 99.48, 105.43, 110.47, 122.13, 127.32, 128.26, 128.35, 128.70, 131.26, 134.65, 136.10, 155.10, 162.52, 163.08, 164.95, 165.93

IR (v_{max} cm^{-1}): 501, 532, 694, 825, 871, 1010, 1118, 1273, 1450, 1604, 1712, 2623, 2870, 2947, 3348

MS m/z (mTOF, ESI+): 471.1777 ($C_{27}H_{28}NaO_6$, M+Na)
449.1957 ($C_{27}H_{29}O_6$, M+H)

i24: **4-((Benzyloxy)carbonyl)phenyl 2-methoxy-4-hexyloxybenzoate**

Quantities used: ***i18*** (1.0 g, 3.9 mmol), benzyl 4-hydroxybenzoate (1.36 g, 5.9 mmol), EDAC (1.13 g, 5.9 mmol), DMAP (6 mg, 50 µmol) and DCM (50 ml). The general Steglich esterification protocol was followed. Column chromatography over silica with 17:3 DCM/hexane as the eluent gave ***i23*** as a viscous oil.

Yield: 0.6 g (33%)

^{1}H NMR (400 MHz, $CDCl_3$): 0.85 (3H, t, *J* = 7.0, CH_3), 1.24 – 1.34 (4H, m, CH_2CH_2), 1.41 (2H, quint, *J* = 7.0, CH_2), 1.74 (2H, quint, *J* = 7.0, CH_2) 3.86 (3H, s, CH_3O), 4.01 (2H, quart, *J* = 7.0, CH_2O), 5.29 (2H, s, Bn), 6.45 (1H, d, *J* = 2.1, Ar), 6.47 (1H, dd, *J* = 2.1, *J* = 8.9, Ar) 7.20 (2H, ddd, *J* = 2.1, *J* = 2.4, *J* = 8.9, Ar), 7.25 - 7.40 (5H, m, Bn), 7.99 (1H, d, *J* = 8.9, Ar), 8.05 (2H, ddd, *J* = 2.1, *J* = 2.4, *J* = 8.9 Ar)

13C NMR (100.5 MHz, CDCl3): 14.12, 22.67, 25.74, 29.15, 31.62, 56.09, 66.84, 68.51, 99.48, 105.44, 110.40, 122.12, 127.32, 128.26, 128.35, 128.70, 131.26, 134.64, 136.09, 155.10, 162.52, 163.08, 164.95, 165.93

IR (ν_{max} cm^{-1}): 509, 586, 640, 694, 756, 802, 840, 887, 1002, 1111, 1195, 1242, 1465, 1566, 1597, 1720, 2862, 2947

MS m/z (mTOF, ESI+): 485.1937 ($C_{27}H_{30}NaO_6$, M+Na)
463.2118 ($C_{28}H_{31}O_6$, M+H)

i28: **4-(4-Butyloxy-2-methoxybenzoyloxy)benzoic acid**

Quantities used: ***i22*** (100 mg, 0.229 mmol), 10% palladium on carbon (10 mg), THF (25 ml) and ethanol (10 ml). The general hydrogenation procedure was followed. Following recrystallisation from ethanol, compound ***i28*** was obtained as white plates.

Yield: 50 mg (63%)

^{1}H NMR (400 MHz, DMSO): 0.95 (3H, t, *J* = 6.4, CH_3), 1.46 (2H, sext, *J* = 6.4, CH_2), 1.74 (2H, quint, *J* = 6.4, CH_2), 3.86 (3H, s, OCH_3), 4.02 (2H, t, *J* = 6.4, CH_2O), 6.51 – 6.55 (2H, m, Ar), 7.20 (2H, ddd, *J* = 1.8, *J* = 2.4, *J* = 8.5, Ar), 7.96 (1H, d, *J* = 9.2, Ar), 8.01 (2H, ddd, *J* = 1.8, *J* = 2.4, *J* = 8.5, Ar)

IR (ν_{max} cm^{-1}): 547, 640, 756, 825, 941, 995, 1141, 1195, 1296, 1419, 1465, 1566, 1681, 1743, 2322, 2546, 2654, 2870, 2954

MS m/z (mTOF, ESI+): 367.1150 ($C_{19}H_{20}NaO_6$, M+Na),
345.1332 ($C_{19}H_{21}O_6$, M+H)

i29: **4-(4-Pentyloxy-2-methoxybenzoyloxy)benzoic acid**

Quantities used: ***i23*** (500 mg, 1.116 mmol), 10% palladium on carbon (50 mg) and THF (90 ml), ethanol (10 ml). The general hydrogenation procedure was followed. Following recrystallisation from ethanol, compound ***i29*** was obtained as white plates.

Yield: 380mg (95%)

^{1}H NMR (400 MHz, DMSO):	0.94 (3H, t, *J* = 7.3, CH_3), 1.36 – 1.50 (4H, m, CH_2CH_2), 1.81 (2H, quint, *J* = 7.3, CH_2), 3.90 (3H, s, CH_3O), 4.05 (2H, t, *J* = 7.3, CH_2O), 6.54 – 6.59 (2H, m, Ar), 7.24 (2H, ddd, *J* = 1.8, *J* = 2.8, *J* = 8.9, Ar), 8.01 (1H, d, *J* = 8.5, Ar), 8.06 (2H, ddd, *J* = 1.8, *J* = 2.8, *J* = 8.9, Ar)
IR (ν_{max} cm^{-1}):	547, 640, 756, 833, 879, 948, 987, 1134, 1203, 1296, 1419, 1566, 1681, 1743, 2322, 2546, 2654, 2862, 2947
MS m/z (mTOF, ESI+):	381.1302 ($C_{20}H_{22}NaO_6$, M+Na)<br>359.1483 ($C_{20}H_{23}O_6$, M+H)

i30: **4-(4-Hexyloxy-2-methoxybenzoyloxy)benzoic acid**

Quantities used: ***i24*** (500 mg, 1.08 mmol), 10% palladium on carbon (50 mg) and THF (90 ml), ethanol (10 ml). The general hydrogenation procedure was followed. Following recrystallisation from ethanol, compound ***i30*** was obtained as white plates.

Yield: 350mg (87%)

^{1}H NMR (400 MHz, DMSO): 0.85 (3H, t, *J* = 7.0, CH_3), 1.27 – 1.33 (4H, m, CH_2CH_2), 1.41 (2H, quint, *J* = 7.0, CH_2), 1.73 (2H, quint, *J* = 7.0, CH_2), 3.84 (3H, s, MeO), 4.02 (2H, t, *J* = 7.0, CH_2O), 6.56 (1H, dd, *J* = 2.1, *J* = 8.9, Ar), 6.59 (1H, d, *J* = 2.1, Ar), 7.24 (2H, d, *J* = 8.5, Ar), 7.91 (1H, d, *J* = 8.9, Ar), 7.98 (2H, d, *J* = 8.5, Ar)

IR (v_{max} cm^{-1}): 547, 640, 756, 833, 887, 948, 1002, 1149, 1195, 1288,1419, 1566, 1604, 1681, 1728, 2538, 2862, 2939

MS m/z (mTOF, ESI+): 395.1462 ($C_{21}H_{24}NaO_6$, M+Na),
373.1639 ($C_{21}H_{25}O_6$, M+H)

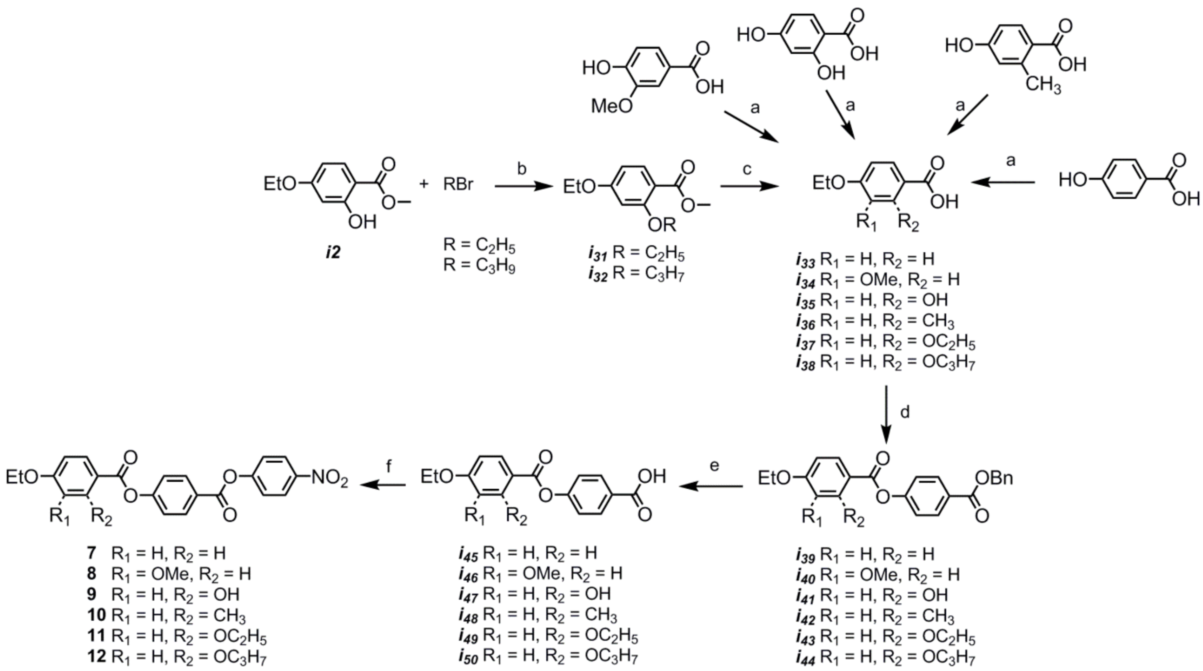

a... (i) EtBr, KOH, H_2O, EtOH
(ii)
b... K_2CO_3, NaI, Acetone
c... (i) LiOH, H_2O, MeOH
(ii) HCl, H_2O
d... Benzyl 4-hydroxybenzoate, EDAC, DMAP, DCM
e... H_2, Pd/C, THF
f... 4-Nitrophenol, EDAC, DMAP, DCM

Scheme 2

i31: **Methyl 2,4-diethoxybenzoate**

Quantities used: ***i2*** (2 g, 10.2 mmol), ethyliodide (1.3 g, 0.91 ml, 12.2 mmol), potassium carbonate (2.9 g, 20.4 mmol), potassium iodide (100 mg) and acetone (30 ml).The general Williamson etherification protocol was followed. The product was isolated from the crude residue by gradient elution column chromatography over silica with DCM moving to 1:1 DCM/methanol giving ***i31*** as a viscous oil.

Yield: 1.6 g (72%)

^{1}H NMR (400 MHz, $CDCl_3$): 1.33 (3H, t, *J* = 7.0, CH_3), 1.38 (3H, t, *J* = 7.0, CH_3), 3.76 (3H, s, CH_3O), 3.93 - 4.03 (4H, m, *J* = 7.0, CH_2O), 6.36 - 6.39 (2H, m, Ar), 7.74 (1H, d, *J* = 8.2, Ar)

^{13}C NMR (100.5 MHz, $CDCl_3$): 14.55, 51.91, 63.74, 101.06, 105.22, 107.89, 131.15, 163.72, 164.98, 170.40

IR (v_{max} cm^{-1}): 516, 655, 702, 771, 810, 910, 972, 1041, 1111, 1211, 1365, 1435, 1504 1573, 2129, 2947, 2970, 3016, 3456

MS m/z (mTOF, ESI+): 247.0946 ($C_{12}H_{16}NaO_4$, M+Na)
225.1123 ($C_{12}H_{17}O_4$, M+H)

i32: **Methyl 2-propoxy-4-ethoxybenzoate**

Quantities used: ***i2*** (2.0 g, 10.2 mmol), 1-bromopropane (1.5 g, 1.11 ml, 12.2 mmol), potassium carbonate (2.9 g, 20.4 mmol), potassium iodide (100 mg) and acetone (30 ml). The general Williamson etherification protocol was followed. The product was isolated from the crude residue by gradient elution column chromatography over silica with DCM moving to 1:1 DCM/methanol giving compound ***i32*** as a viscous oil.

Yield:	1.8 g (75%)
^{1}H NMR (400 MHz, $CDCl_3$):	1.00 (3H, t, *J* = 7.0, CH_3), 1.33 (3H, t, *J* = 7.0, CH_3), 1.78 (2H, sextet, *J* = 7.0, CH_2), 3.76 (3H, s, CH_3O), 3.88 (2H, t, *J* = 7.0, CH_3O), 3.97 (2H, quart, *J* = 7.02, CH_2), 6.38 (2H, m, Ar), 7.74 (1H, m, Ar).
^{13}C NMR (100.5 MHz, $CDCl_3$):	10.41, 14.55, 22.39, 51.41, 63.55, 70.19, 100.13, 104.90, 112.20, 133.64, 160.73, 163.40, 166.25
IR (v_{max} cm^{-1}):	516, 586, 663, 771, 902, 964, 1026, 1126, 1203, 1372, 1435, 1504, 1566, 1597, 1681, 1735, 2129, 2877, 3456, 3741

i33: 4-Ethoxybenzoic acid

Bromoethane (23.7 g, 0.217 mol) was added to a stirred solution of 4-Hydroxybenzoic acid (30.0 g, 0.217 mol) and sodium hydroxide (17.4 g, 0.435 mol) in ethanol (300 ml) and water (150 ml) at 60 °C. After 16h the solution was cooled, poured onto crushed ice and acidified with 36% hydrochloric acid, affording a voluminous white precipitate. The precipitate was collected by filtration and recrystallised from ethanol, furnishing ***i33*** as colourless plates.

Yield: 31.5 g (87%)

Melting Point (°C): 198.2

^{1}H NMR (400 MHz, DMSO): 1.33 (3H, t, *J* = 7.0, CH_3), 4.01 (2H, quart, *J* = 7.0, CH_2O), 6.87 (2H, d, *J* = 8.9, Ar), 7.85 (2H, d, *J* = 8.9, Ar)

^{13}C NMR (100.5 MHz, DMSO): 14.42, 63.26, 113.79, 122.79, 131.25, 162.09, 167.14

IR (ν_{max} cm^{-1}): 547, 640, 694, 771, 848, 925, 1041, 1118, 1172, 1257, 1296, 1388, 1427, 1512, 1604, 1666, 2654, 2816, 2893, 2939

MS m/z (mTOF, ESI+): 167.0701 (100%, $C_9H_{11}O_3$, M+H),

***i34*: 4-Ethoxy-3-methoxybenzoic acid**

Quantities used: 4-hydroxy-3-methoxybenzoic acid (10.0 g , 59.5 mmol), bromoethane (14.27 g, 9.7 ml, 130.9 mmol), potassium hydroxide (10.0 g, 178.5 mmol), ethanol (100 ml) and water (30 ml). The experimental procedure was as described in the preparation of compound ***i33***.Recrystallisation from ethanol/water gave compound ***i34*** as off white needles.

Yield: 11.3 g (97 %)

^{1}H NMR (400 MHz, DMSO): 1.33 (3H, t, *J* = 6.7, CH_3), 3.78 (3H, s, OCH_3), 4.04 (2H, q, *J* = 6.7, CH_2O), 6.86 (1H, d, *J* = 8.5, Ar), 7.40 (1H, d, *J* = 1.8, Ar), 7.51 (1H, dd, *J* = 1.8, *J* = 8.5, Ar)

IR (ν_{max} cm^{-1}): 524, 640, 717, 817, 871, 925, 1026, 1111, 1141, 1226, 1273, 1342, 1419, 1458, 1512, 1581, 1674, 1874, 2538, 2939

MS (m/z, ESI+): 209.0622 ($C_{10}H_{12}NaO_4$, M+Na)
197.0803 (10%, $C_{10}H_{13}O_4$. M+H)

i35: **4-Ethoxy-2-hydroxybenzoic acid**

Quantities used: 2,4-dihydroxybenzoic acid (30.0 g, 0.194 mol), bromoethane (42.2 g, 0.388 mol), sodium hydroxide (15.52 g, 0.388 mol), ethanol (300 ml) and water (150 ml). The experimental procedure was as described in the preparation of compound ***i33***. Recrystallisation from ethanol gave ***i35*** as colourless needles.

Yield:	34.5 g (98%)
Melting Point (°C):	152.4 - 153.3
^{1}H NMR (400 MHz, DMSO):	0.98 (3H, t, *J* = 7.0, CH_3), 3.61 (2H, quart, *J* = 7.0, CH_2O), 6.42 (1H, d, *J* = 2.1, Ar), 6.45 (1H, d, *J* = 2.1, 8.9, Ar), 7.79 (1H, d, *J* = 8.9, Ar), 10.59 (1H, s, OH)
^{13}C NMR (100.5 MHz, DMSO):	14.45, 63.59, 101.05, 105.46, 107.34, 131.61, 163.43, 164.41, 171.90
IR (v_{max} cm^{-1}):	524, 594, 678, 771, 894, 979, 1033, 1103, 1219, 1365, 1442, 1573, 1620, 1735, 2553, 2700, 2870, 2947, 3194, 3456
MS m/z (mTOF, ESI+):	205.0469 ($C_9H_{10}NaO_4$, M+Na), 183.0652 (100%, $C_9H_{11}O_4$, M+H), 162.05, 137.02

i37: **2,4-Diethoxybenzoic acid**

Quantities used: ***i31*** (0.8 g, 3.57 mmol), lithium hydroxide (0.26 g, 10.7 mmol), methanol (20 ml) and water (20 ml). The general ester hydrolysis protocol was employed. Compound ***i37*** was obtained as a white solid following acidification.

Yield:	0.7 g (95%)
Melting Point (°C):	99.4
^{1}H NMR (400 MHz, DMSO):	1.31 (6H, t, *J* = 7.0, CH_3), 40.4 (4H, quart, *J* = 7.0, CH_2O), 6.49 (1H, dd, *J* = 2.4, *J* = 8.5, Ar), 6.51 (1H, d, *J* = 2.4, Ar), 7.67 (1H, d, *J* = 8.5, Ar)
^{13}C NMR (400 MHz, DMSO):	14.38, 63.35, 64.02, 99.94, 105.38, 112.63, 133.14, 159.66, 162.76, 166.32

i38: **2-Propoxy-4-ethoxybenzoic acid**

Quantities used: ***i32*** (0.8 g, 3.36 mmol), lithium hydroxide (0.24 g, 10.1 mmol), methanol (20 ml) and water (20 ml). The general ester hydrolysis protocol was employed. Compound ***i38*** was obtained as a white solid following acidification.

Yield: 0.6 g (74%)

^{1}H NMR (400 MHz, DMSO): 1.00 (3H, t, J = 7.0, CH_3), 1.32 (3H, t, J = 7.0, CH_3), 1.70 (2H, sextet, J = 7.0, CH_2), 3.94 (2H, t, J = 7.0, CH_2O), 4.04 (2H, quart, J = 7.0, CH_2O), 6.46 - 6.48 (2H, m, Ar), 7.66 (1H, d, J = 8.5, Ar).

^{13}C NMR (100.5 MHz, DMSO): 10.37, 14.44, 21.99, 63.34, 69.77, 99.81, 105.41, 112.59, 133.22, 159.93, 162.85, 166.45

i39: **Benzyl 4-(4-ethoxybenzoyloxy)benzoate**

Quantities used: ***i31*** (5 g, 30.1 mmol), benzyl 4-hydroxybenzoate (6.2 g, 27.4 mmol), DCC (6.2 g, 30.1 mmol), DMAP (0.3 g, 2.7 mmol) and DCM (100 ml).The general Steglich esterification protocol was followed. Column chromatography over silica with 1:1 DCM/hexane as the eluent gave ***i39*** as a white solid.

Yield:	7.6 g (74%)
Melting Point (°C):	105.5
^{1}H NMR (400 MHz, $CDCl_3$):	1.45 (3H, t, *J* = 7.0, CH_3), 4.09 (2H, quart, *J* = 7.0, CH_2O), 5.36 (2H, s, Bn), 6.95 (2H, ddd, *J* = 2.2, *J* = 2.6, *J* = 9.3, Ar), 7.28 (2H, ddd, *J* = 1.8, *J* = 2.6, *J* = 8.5, Ar), 7.33 - 7.47 (5H, m, Bn), 8.11 - 8.14 (2H, ddd, *J* = 2.2, *J* = 2.6, *J* = 9.3, Ar), 8.13 - 8.16 (2H, ddd, *J* = 1.8, *J* = 2.6, *J* = 8.5, Ar)
^{13}C NMR (100.5 MHz, $CDCl_3$):	14.59, 63.79, 66.73, 114.29, 121.02, 121.81, 127.45, 128.11, 128.23, 128.56, 131.24, 132.34, 135.91, 154.85, 163.49, 164.27, 165.65
IR (v_{max} cm^{-1}):	516, 686, 732, 756, 840, 894, 972, 1064, 1095, 1157, 1211, 1373, 1442, 1504, 1604, 1728, 2870, 2939, 2970, 3016, 3456
MS m/z (mTOF, ESI+):	394.1649 ($C_{23}H_{24}NO_5$, M+NH_4)<br>377.1392 ($C_{23}H_{21}O_5$, M+H)

i40: **4-((Benzyloxy)carbonyl)phenyl 4-ethoxy-3-methoxybenzoate**

Quantities used: ***i34*** (2 g, 10.204 mmol), benzyl 4-hydroxybenzoate (5.12 g, 22.44 mmol), EDAC (4.29 g, 22.44 mmol), DMAP (5 mg, 82.5 µmol) and DCM (50 ml). The general Steglich esterification protocol was employed. Silica gel column chromatography with 1:1 DCM/hexanes as the eluent afforded compound the title compound as a viscous oil

Yield: 3.6 g (87%)

^{1}H NMR (400 MHz, $CDCl_3$): 1.50 (3H, t, *J* = 7.3, CH_3), 3.93 (3H, s, OCH_3), 4.18 (2H, q, *J* = 7.3, CH_2O), 5.36 (2H, s, Bn), 6.92 (1H, d, *J* = 8.8, Ar), 7.26 (2H, ddd, *J* = 1.8, *J* = 2.4, *J* = 8.4, Ar), 7.33 – 7.45 (5H, m, Bn), 7.63 (1H, d, *J* = 1.8, Ar), 7.82 (1H, dd, *J* = 1.8, *J* = 8.4, Ar), 8.13 (2H, ddd, *J* = 1.8, *J* = 2.4, *J* = 8.8, Ar)

IR (v_{max} cm^{-1}): 509, 578, 686, 748, 871, 902, 1018, 1072, 1203, 1265, 1373, 1450, 1512, 1597, 1720, 2939, 2970, 3032

MS m/z (mTOF, ESI+): 429.1315 (100%, $C_{24}H_{22}NaO_6$, M+Na) 407.1489 ($C_{24}H_{23}O_6$, M+H)

i41: **4-(Benzyloxycarbonyl)phenyl 4-ethoxy-2-hydroxybenzoate**

Quantities used: ***i35*** (10 g, 54.9 mmol), benzyl 4-hydroxybenzoate (11.4 g, 49.9 mmol), DCC (10.3 g, 49.9 mmol), DMAP (6.1 g, 49.9 mmol), DCM (50 ml) and stirred for 24h. The general Steglich esterification protocol was followed. The title compound was isolated as a white solid from the crude reaction residues by column chromatography over silica gel with ethyl acetate as the eluent.

Yield:	9.9 g (51%)
Melting Point (°C):	94.5
^{1}H NMR (400 MHz, $CDCl_3$):	1.42 (3H, t, *J* = 7.0, CH_3), 4.06 (2H, quart, *J* = 7.0, CH_2O), 5.36 (2H, s, Bn), 6.46 (1H, d, *J* = 1.8, Ar), 6.50 (1H, dd, *J* = 1.8, *J* = 8.9, Ar), 7.26 (2H, d, *J* = 8.5, Ar), 7.33 - 7.48 (5H, m, Bn), 7.92 (1H, d, *J* = 8.9, Ar), 8.14 (2H, d, *J* = 8.5, Ar), 10.54 (1H, s, OH)
^{13}C NMR (100.5 MHz, $CDCl_3$):	14.55, 63.98, 66.87, 101.22, 104.19, 108.61, 121.84, 127.99, 128.19, 128.31, 128.61, 131.38, 131.67, 135.86, 153.94, 164.60, 165.49, 165.85, 168.09
IR (v_{max} cm^{-1}):	501, 578, 686, 725, 763, 817, 887, 979, 1041, 1111, 1165, 1195, 1249, 1357, 1458, 1504, 1581, 1674, 1712, 1921, 2607, 2939, 3070, 3186
MS m/z (mTOF, ESI+):	393.1328 (100%, $C_{23}H_{20}O_6$, M+H),

i42: **4-((Benzyloxy)carbonyl)phenyl 4-ethoxy-2-methylbenzoate**

Quantities used: ***i36*** (1.0 g, 5.56 mmol), benzyl 4-hydroybenzoate (1.6 g, 6.94 mmol), EDAC (1.33 g, 6.94 mmol), DMAP (50 mg, 0.426 mmol) and DCM (50 ml). The general Steglich esterification protocol was employed. Column chromatography over silica with 1:1 DCM/hexane as the eluent gave ***i42*** as a white solid.

Yield: 1.4 g (65%)

^{1}H NMR (400 MHz, $CDCl_3$): 1.43 (3H, t, *J* = 7.0, CH_3), 2.64 (3H, s, Ar-CH_3), 4.08 (2H, q, *J* = 7.0, CH_2O), 5.36 (2H, s, Bn), 6.77 – 6.82 (2H, m, Ar), 7.26 (2H, ddd, *J* = 1.8, *J* = 2.6, *J* = 8.8, Ar), 7.30 – 7.47 (5H, m, Bn), 8.11 – 8.18 (3H, m, Ar)

IR (v_{max} cm^{-1}): 640, 686, 717, 763, 864, 1026, 1103, 1195, 1242, 1404, 1450, 1496, 1558, 1604, 1712, 2878, 3070

MS m/z (mTOF, ESI+): 413.1356 ($C_{24}H_{22}NaO_5$, M+Na),
391.1536 (100%, $C_{24}H_{23}O_5$, M+H)

i43: **4-(Benzyloxycarbonyl)phenyl 2,diethoxybenzoate**

Quantities used: ***i37*** (0.6 g, 2.86 mmol), benzyl 4-hydroybenzoate (0.71 g, 3.14 mmol), EDAC (0.82 g, 4.29 mmol), DMAP (52 mg, 0.429 mmol) and DCM (25 ml). The general Steglich esterification protocol was employed. Column chromatography over silica with 1:1 DCM/hexane as the eluent gave ***i43*** as a white solid.

Yield:	0.8 g (68%)
^{1}H NMR (400 MHz, $CDCl_3$):	1.41 - 1.46 (6H, m, *J* = 7.0, CH_3), 4.05 - 4.13 (4H, m, *J* = 7.0, CH_2O), 5.35 (2H, s, Bn), 6.48 (1H, d, *J* = 2.1, Ar), 6.51 (1H, dd, *J* = 2.1, *J* = 8.9, Ar), 7.26 (2H, ddd, *J* = 1.8, *J* = 2.4, *J* = 8.9, Ar), 7.30 - 7.44 (5H, m, Bn), 8.01 (1H, d, *J* = 8.9, Ar), 8.11 (2H, ddd, *J* = 1.8, *J* = 2.4, *J* = 8.9, Ar)
^{13}C NMR (100.5 MHz, $CDCl_3$):	14.64, 63.85, 64.56, 66.69, 100.26, 105.32, 110.67, 122.00, 127.19, 128.11, 128.22, 128.58, 131.19, 134.46, 135.99, 155.05, 161.71, 163.20, 164.45, 165.81
IR (v_{max} cm^{-1}):	501, 578, 740, 810, 879, 1010, 1103, 1195, 1273, 1373, 1435, 1566, 1604, 1720, 1743, 2113, 2499, 2576, 2638, 2939, 2978, 3024, 3086, 3646, 3741
MS m/z (mTOF, ESI+):	443.14 (M+Na),<br>421.1645 (100%, $C_{25}H_{26}O_6$, M+H)

i44: **4-(Benzyloxycarbonyl)phenyl 4-ethoxy-2-propoxybenzoate**

Quantities used: ***i38*** (0.6 g, 2.68 mmol), benzyl 4-hydroybenzoate (0.67 g, 2.95 mmol), EDAC (0.77 g, 4.02 mmol), DMAP (49mg, 0.4 mmol) and DCM (25 ml). The general Steglich esterification protocol was employed. Column chromatography over silica with 1 DCM as the eluent gave ***i44*** as a white solid.

Yield: 0.78 g (67%)

^{1}H NMR (400 MHz, $CDCl_3$): 0.97 (3H, t, *J* = 7.0, CH_2), 1.37 (3H, t, *J* = 7.0, CH_2), 1.78 (2H, sextet, *J* = 7.0, CH_2), 3.92 (2H, t, *J* = 7.0, CH_2O), 4.03 (2H, quart, *J* = 7.0, CH_2O), 5.29 (2H, s, Bn), 6.42 (1H, d, *J* = 2.4, Ar), 6.44 (1H, dd, *J* = 2.4, *J* = 8.5, Ar), 7.20 (2H, ddd, *J* = 1.8, *J* = 3.1, *J* = 8.5, Ar), 7.24 - 7.38 (5H, m, Bn), 7.95 (1H, d, *J* = 8.5, Ar), 8.05 (2H, ddd, *J* = 1.8, *J* = 3.1, *J* = 8.5, Ar).

^{13}C NMR (100.5 MHz, $CDCl_3$): 10.58, 14.63, 22.46, 63.82, 66.68, 70.33, 100.07, 105.25, 110.63, 121.98, 127.16, 128.10, 128.20, 128.57, 131.20, 134.53, 135.99, 155.07, 161.78, 163.35, 164.44, 165.79

IR (v_{max} cm^{-1}): 509, 594, 624, 694, 725, 756, 802, 864, 1002, 1111, 1195, 1234, 1373, 1435, 1496, 1566, 1604, 1735, 2877, 2939, 2970, 3086, 3456

MS m/z (mTOF, ESI+): 457.1614 (M+Na),
435.1809 (100%, $C_{26}H_{27}O6$, M+H)

i45: **4-(4-Ethoxybenzoyloxy)benzoic acid (61)**

Quantities used: ***i39*** (7.0 g, 18.6 mol), 10% palladium on carbon (0.1 g) and THF (125 ml). The general hydrogenation protocol was followed. Recrystallisation from ethanol gave compound ***i45*** as white needles.

Yield:	5.2 g (98%)
Phase Transitions (°C):	Cr 223.2 N 263.0 Iso
^1H NMR (400 MHz, DMSO):	1.34 (3H, t, *J* = 7.0, CH_3), 4.11 (2H, quart, *J* = 7.0, CH_2O), 7.08 (2H, d, *J* = 8.9, Ar), 7.36 (2H, d, *J* = 8.5, Ar), 8.01 (2H, d, *J* = 8.5, Ar), 8.05 (2H, d, *J* = 8.9, Ar), 13.04 (1H, br s, COOH)
^{13}C NMR (100.5 MHz, DMSO):	14.44, 63.71, 114.67, 120.42, 122.22, 128.33, 130.88, 132.14, 154.20, 163.20, 163.82, 168.62
IR (v_{max} cm^{-1}):	501, 624, 756, 840, 918, 1010, 1041, 1111, 1157, 1211, 1365, 1427, 1512, 1604, 1681, 1735, 2121, 2322, 2561, 2661, 2870, 2947, 3001, 3464
MS m/z (mTOF, ESI+):	309.0727 (M+Na)<br>287.0914 ($C_{16}H_{15}O_5$, M+H)

i46: **4-((4-Ethoxy-3-methoxybenzoyl)oxy)benzoic acid**

Quantities used: Compound ***i40*** (2.2 g, 5.42 mmol), 10% palladium on carbon (20 mg), THF (60 ml) and ethanol (10 ml). The general hydrogenation protocol was followed. The crude material was recrystallised from ethanol giving ***i46*** as colourless needles.

Yield: 1.7 g (99%)

^{1}H NMR (400 MHz, DMSO): 1.36 (3H, t, *J* = 7.0, CH_3), 3.83 (3H, s, OCH_3), 4.12 (2H, q, *J* = 7.0, CH_2O), 7.09 (1H, d, *J* = 8.4, Ar), 7.33 (2H, ddd, *J* = 1.8, *J* = 2.6, *J* = 8.8, Ar), 7.56 (1H, d, *J* = 2.2, Ar), 7.74 (1H, dd, *J* = 2.2, *J* = 8.4, Ar), 8.00 (2H, ddd, *J* = 1.8, *J* = 2.6, *J* = 8.8, Ar)

IR (ν_{max} cm^{-1}): 547, 725, 756, 810, 871, 902, 1026, 1072, 1134, 1203, 1265, 1411, 1512, 1589, 1681, 1728

i47: **4-(4-Ethoxy-2-hydroxybenzoyloxy)benzoic acid**

Quantities used: ***i42*** (9.0 g, 22.9 mmol), 10% palladium on carbon (0.1 g), THF (40 ml). The general hydrogenation protocol was followed. Recrystallisation from ethanol gave *i**47*** as colourless needles.

Yield:	6.4 g (92%)
Phase Transitions (°C):	Cr 197.0 N 225 Decomposes
^{1}H NMR (400 MHz, DMSO):	1.33 (3H, t, *J* = 7.0, CH_3), 4.05 (2H, quart, *J* = 7.0, CH_2O), 6.44 (1H, d, *J* = 2.4, Ar), 6.49 (1H, dd, *J* = 2.4, *J* = 8.9, Ar), 7.29 (2H, ddd, *J* = 1.8, *J* = 2.4, *J* = 8.5, Ar), 7.88 (1H, d, *J* = 8.9, Ar), 7.99 (2H, ddd, *J* = 1.8, *J* = 2.4, *J* = 8.5, Ar), 10.36 (1H, s, OH), 12.81 (1H, broad s, COOH)
^{13}C NMR (100.5 MHz, DMSO):	12.63, 61.98, 99.58, 102.55, 106.28, 120.12, 126.99, 129.12, 130.12, 151.63, 161.82, 163.59, 164.86, 165.37
IR (v_{max} cm^{-1}):	501, 547, 640, 756, 840, 871, 933, 979, 1033, 1141, 1195, 1257, 1427, 1504, 1573, 1681, 2322, 2546, 2661, 2816, 2893, 2947, 3070, 3294
MS m/z (mTOF, ESI+):	325.0679 ($C_{16}H_{17}NaO_6$, M+Na),<br>303.0863 (100%, $C_{16}H_{18}O_6$, M+H),

i48: **4-((4-Ethoxy-2-methylbenzoyl)oxy)benzoic acid (138)**

Quantities used: ***i42*** (0.5 g, 1.28 mmol), 10% palladium on carbon (20 mg), THF (20 ml) and ethanol (20 ml). The general hydrogenation protocol was followed. Recrystallisation from ethanol gave ***i48*** as colourless needles.

Yield:	0.32 g (83%)
^{1}H NMR (400 MHz, DMSO):	1.23 (3H, t, *J* = 7.0, CH_3), 2.43 (3H, s, Ar-CH_3), 3.98 (2H, q, *J* = 7.0, CH_2O), 6.72 – 6.77 (2H, m , Ar), 7.19 (2H, ddd, *J* = 2.2, *J* = 2.6, *J* = 8.8, Ar), 7.88 (2H, ddd, *J* = 2.2, *J* = 2.6, *J* = 8.8, Ar), 7.95 (1H, d, *J* = 9.5, Ar)
IR (v_{max} cm^{-1}):	686, 756, 817, 964, 1002, 1041, 1118, 1157, 1203, 1256, 1350, 1419, 1519, 1604, 1712, 1928, 2985, 3086

i49: **4-(2,4-Diethoxybenzoyloxy)benzoic acid**

Quantities used: ***i39*** (0.7 g, 1.66 mmol), 10% palladium on carbon (10 mg), THF (20 ml) and ethanol (20 ml). The general hydrogenation protocol was followed. Recrystallisation from ethanol gave ***i49*** as colourless needles.

Yield:	0.48 g (87%)
Melting Point (°C):	177.4
^{1}H NMR (400 MHz, DMSO):	1.34 (6H, t, *J* = 6.7, CH_3), 4.08 (4H, quart, *J* = 6.7, CH_2O), 6.50 - 6.53 (2H, m, Ar), 7.20 (2H, d, *J* = 8.9, Ar), 7.87 (1H, d, *J* = 9.5, Ar), 7.97 (2H, d, *J* = 8.9, Ar)
^{13}C NMR (100.5 MHz, DMSO):	13.04, 62.17, 62.78, 98.49, 104.20, 108.73, 120.32, 126.62, 129.35, 132.51, 152.96, 159.73, 161.35, 162.74, 165.20
IR (ν_{max} cm^{-1}):	509, 547, 663, 686, 756.1, 817, 995, 1111, 1149, 1203, 1373, 1427, 1504, 1566, 1604, 1674, 1743, 2314, 2546, 2661, 2870, 2939.09, 2978, 3464
MS m/z (mTOF, ESI+):	353.0992 ($C_{18}H_{18}NaO_6$, M+Na),<br>331.1175 (100%, $C_{18}H_{19}O_6$, M+H)

i50: **4-(4-Ethoxy-2-propoxybenzoyloxy)benzoic acid**

Quantities used: ***i44*** (0.65 g, 1.49 mmol), 10% palladium on carbon (10 mg), THF (20 ml) and ethanol (20 ml). The general hydrogenation protocol was followed. Recrystallisation from ethanol gave ***i50*** as colourless needles.

Yield:	0.44 g (85%)
Melting Point (°C):	191.9
^{1}H NMR (400 MHz, DMSO):	0.97 (3H, t, *J* = 7.0, CH_3), 1.33 (3H, t, *J* = 7.0, CH_3), 1.71 (2H, sextet, *J* = 7.0, CH_2), 4.01 (2H, t, *J* = 7.0, CH_2O), 4.13 (2H, t, *J* = 7.0, CH_2O), 6.61 (1H, dd, *J* = 2.1, *J* = 8.9, Ar), 6.64 (1H, d, *J* = 2.1, Ar), 7.31 (2H, ddd, *J* = 1.8, *J* = 2.4, *J* = 8.5, Ar), 7.90 (1H, d, *J* = 8.9, Ar), 8.00 (2H, ddd, *J* = 1.8, *J* = 2.4, *J* = 8.5, Ar)
^{13}C NMR (100.5 MHz, DMSO):	10.47, 14.46, 22.00, 63.73, 69.78, 99.97, 106.04, 109.90, 122.20, 128.11, 130.88, 134.00, 154.37, 161.16, 162.86, 164.14, 166.70
IR (v_{max} cm^{-1}):	509, 763, 825, 879, 956, 995, 1111, 1141, 1195, 1296.16, 1373, 1427, 1504, 1566, 1604, 1674, 1743, 2314, 2546, 2661, 2862, 2970, 2463
MS m/z (mTOF, ESI+):	367.1145 ($C_{19}H_{20}NaO_6$, M+Na),<br>345.1323 (100%, $C_{19}H_{21}O_6$, M+H)

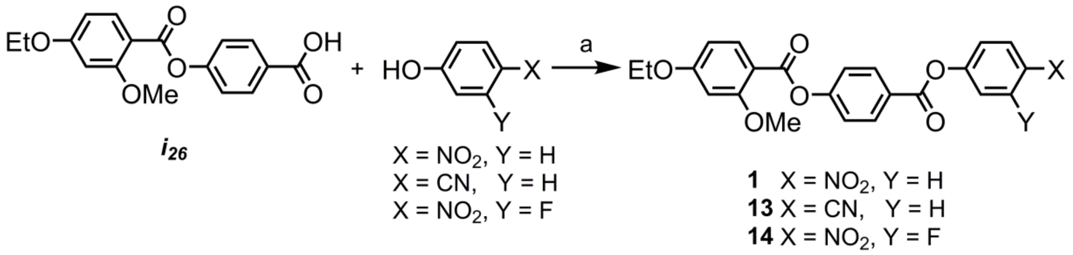

Scheme 3

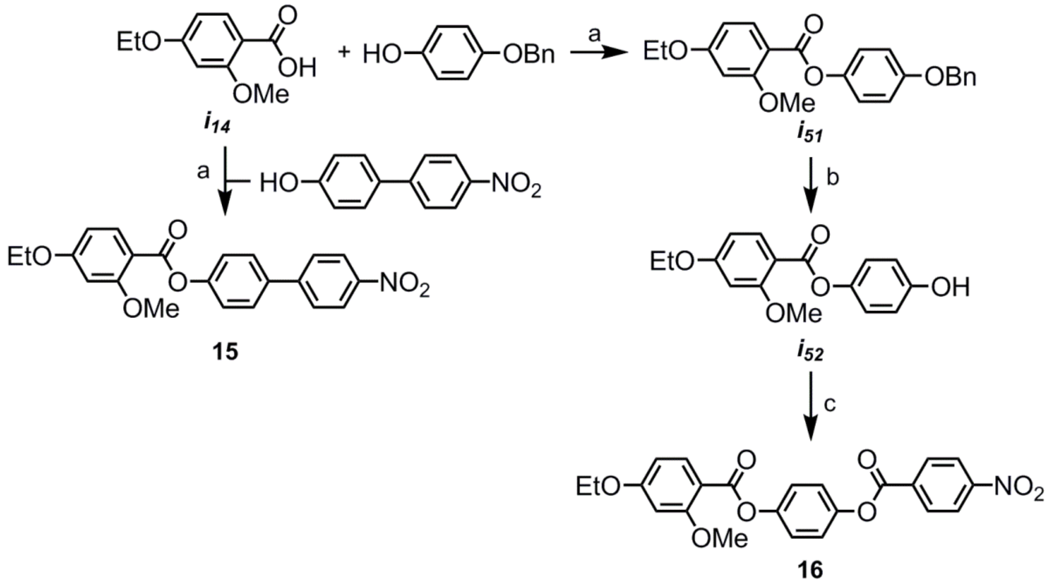

a... EDAC, DMAP, DCM
b... H_2, Pd/C, THF
c... 4-Nitrobenzoic acid, EDAC, DMAP, DCM

Scheme 4

i51: **4-(Benzyloxy)phenyl 4-ethoxy-2-methoxybenzoate**

Quantities used: ***i14*** (500 mg, 2.551 mmol), 4-benzyloxyphenol (612 mg, 3.06 mmol), EDAC (584 mg, 3.06 mmol), DMAP (6 mg, 50 µmol) and DCM (50 ml). The standard Steglich esterification protocol was followed. Column chromatography over silica with 17:3 DCM/hexane as the eluent gave ***i51*** as a viscous oil.

Yield: 0.82 g (85%)

^{1}H NMR (400 MHz, $CDCl_3$): 1.38 (3H, t, *J* = 7.0, CH_3), 3.83 (3H, s, OCH_3), 4.04 (2H, q, *J* = 7.0, CH_2O), 4.99 (2H, s, Bn), 6.44 – 6.49 (2H, m, Ar), 6.91 (2H, ddd, *J* = 2.1, *J* = 3.7, *J* = 9.2, Ar), 7.04 (2H, ddd, *J* = 2.1, *J* = 3.7, *J* = 9.2, Ar), 7.22 – 7.40 (5H, m, Bn), 7.79 (1H, d, *J* = 8.9, Ar)

MS m/z (ESI+): 401.1339 ($C_{23}H_{22}NaO_5$, M+Na)
379.1525 (100%, $C_{23}H_{23}O_5$, M+H)

i52: **4-Hydroxyphenyl 4-ethoxy-2-methoxybenzoate**

Quantities used: ***i51*** (500 mg, 1.73 mmol), 10% palladium on carbon (20 mg) and THF (25 ml) and ethanol (10 ml). The general hydrogenation protocol was followed. After removal of the solvents *in vacuo* compound ***i52*** was purified by column chromatography with DCM, affording the title compound as a white solid.

Yield: 0.32 g (75%)

^{1}H NMR (400 MHz, $CDCl_3$): 1.43 (3H, t, *J* = 7.0, CH_3), 3.88 (3H, s, OCH_3), 4.09 (2H, q, *J* = 7.0, CH_2O), 6.48 – 6.53 (2H, m, Ar), 6.79 (2H, ddd, *J* = 2.3, *J* = 3.7, *J* = 9.2, Ar), 6.99 (2H, ddd, *J* = 2.3, *J* = 3.7, *J* = 9.2, Ar), 8.02 (1H, d, *J* = 8.4, Ar)

MS m/z (ESI+): 311.0884 (100%, $C_{16}H_{16}NaO_5$, M+Na)
289.1068 ($C_{16}H_{17}O_5$, M+H)

1.4. Tabulated Mixture Data

Sample No.	Masses [mg]		Weight percent		Transition Temperatures		
	cpd 2	cpd 1	cpd 2	cpd1	MP	Nx-N	N-Iso
**cpd 1**	**0**	**1**	**0%**	**100%**	**138.5**	**85.6**	**189**
1.1	0.38	1.54	20%	80%	131	86	184
1.2	0.76	1.16	40%	60%	124	98	194
1.3	1.18	0.78	60%	40%	116	112	188
1.4	1.61	0.39	81%	20%	126	115	199
**cpd 2**	**1**	**0**	**100%**	**0%**	**131**	**128**	**194**

Table SI-1: Masses (mg) weight percent (wt %) and transition temperatures (°C) for binary mixtures (1.**X**) of compounds **1** and **2**. Data plotted in the text as Figure 2a

Sample No.	Masses [mg]		Weight percent		Transition Temperatures		
	cpd 3	cpd 1	cpd 3	cpd1	MP	Nx-N	N-Iso
**cpd 1**	**0**	**1**	**0%**	**100%**	**138.5**	**85.6**	**189**
2.1	0.25	1.99	11%	89%	131.4	78.3	178.6
2.2	0.39	1.64	19%	81%	127.2	68.9	176.1
2.3	0.57	1.39	29%	71%	124.5	63.4	175.5
2.4	0.87	1.17	43%	57%	121.9	51.7	172.3
2.5	1.04	1.13	48%	52%	121.5	-	172.3
2.6	1.31	0.88	60%	40%	119.5	-	170.1
2.7	1.45	0.63	70%	30%	118.5	-	167.6
2.8	1.56	0.38	80%	20%	117.2	-	166.2
2.9	1.80	0.21	90%	10%	119.5	-	163.4
**cpd 3**	**1**	**0**	**100%**	**0%**	**134.4**	**-**	**163.5**

Table SI-2: Masses (mg) weight percent (wt %) and transition temperatures (°C) for binary mixtures (2.**X**) of compounds **1** and 3. Data plotted in the text as Figure 2b

Sample No.	Masses [mg]		Weight percent		Transition Temperatures		
	cpd 7	cpd 1	cpd 7	cpd1	MP	Nx-N	N-Iso
**cpd 1**	**0**	**1**	**0%**	**100%**	**138.5**	**85.6**	**189**
3.1	0.094	10.04	1%	99%	134.1	84.1	183.34
3.2	0.186	9.81	2%	98%	132.7	82.3	185.66
3.3	0.188	4.86	5%	95%	130	80.2	188.54
3.4	0.281	4.74	7%	93%	128.2	78.4	190.13
3.5	0.368	4.57	8%	92%	127.6	77.3	190.47
3.6	0.456	4.44	10%	90%	126.9	-	190.37
3.7	0.916	4.00	21%	79%	135.5	-	205.6
3.8	1.369	3.53	30%	70%	132.0	-	209.4
3.9	1.852	3.12	39%	61%	120.2	-	218.6
3.10	2.282	2.62	49%	51%	128.1	-	231.1
3.11	2.677	2.11	61%	39%	129.6	-	243.6
3.12	3.077	1.64	69%	31%	122.4	-	249.4
3.13	3.621	1.24	80%	20%	144.3	-	262.2
3.14	3.956	0.76	90%	10%	151.6	-	272.4
**cpd 7**	**1**	**0**	**100%**	**0%**	**160.3**	**-**	**276.4**

Table SI-3: Masses (mg) weight percent (wt %) and transition temperatures (°C) for binary mixtures (3.**X**) of compounds **1** and **7**. Data plotted in the text as Figure 6a

	Masses [mg]		Weight percent		Transition Temperatures		
**Sample No.**	**cpd 9**	**cpd 1**	**cpd 9**	**cpd1**	**MP**	**Nx-N**	**N-Iso**
**cpd 1**	**0**	**1**	**0%**	**100%**	**138.5**	**85.6**	**189**
4.1	0.1	10.2	1%	99%	126.2	81.4	187.6
4.2	0.2	10.1	2%	98%	123.7	79.5	178.7
4.3	0.4	10.2	4%	96%	123.5	79.4	181.3
4.4	0.6	10.4	6%	94%	123.1	76.4	181.9
4.5	0.5	5	8%	92%	123.7	75.1	185.3
4.6	1	4.5	10%	90%	120.4	-	181.2
4.7	1.5	4	21%	79%	112.6	-	190.3
4.8	2	3.5	32%	68%	114.8	-	197.4
4.9	2.5	3	40%	60%	123.8	-	201.8
4.10	3	2.5	50%	50%	111.9	-	204.1
4.11	3.5	2	61%	39%	110.0	-	205.2
4.12	4	1.5	70%	30%	139	-	210.8
4.13	4.5	1	81%	19%	148.9	-	216.5
4.14	5	0.5	91%	9%	156.5	-	222.5
**cpd 9**	**1**	**0**	**100%**	**0%**	**163.0**	**-**	**>240***

Table SI-4: Masses (mg) weight percent (wt %) and transition temperatures (°C) for binary mixtures (4.**X**) of compounds **1** and **9**. Data plotted in the text as Figure 6b. * the neat material (**9**) decomposes prior to the N-Iso transition, however the clearing point appears to be around 240 °C

Sample No.	Masses [mg]		Weight percent		Transition Temperatures		
	cpd 10	cpd 1	cpd 10	cpd1	MP	Nx-N	N-Iso
**cpd 1**	**0**	**1**	**0%**	**100%**	**138.5**	**85.6**	**189**
5.1	10	0.5	5%	95%	139.0	80	175
5.2	3.6	0.46	11%	89%	132.0	76.7	181
5.3	4.62	0.94	16%	84%	115.0	73.6	182
5.4	3.7	1.22	25%	75%	111.0	-	188
5.5	1.76	0.98	35%	65%	109.0	-	192.81
5.6	3.12	2.16	40%	60%	115.0	-	193.66
5.7	3.0	3.10	49%	51%	114.0	-	196
5.8	3.6	2.5	60%	40%	117.0	-	200
5.9	1.43	3.67	71%	29%	118.0	-	203.27
5.10	1.26	5.29	80%	20%	121.1	-	205.22
**cpd 10**	**1**	**0**	**100%**	**0%**	**138.8**	**-**	**212.1**

Table SI-5: Masses (mg) weight percent (wt %) and transition temperatures (°C) for binary mixtures (5.**X**) of compounds **1** and **10**. Data plotted in the text as Figure 6c.

Sample No.	Masses [mg]		Weight percent		Transition Temperatures		
	cpd 13	cpd 1	cpd 13	cpd1	MP	Nx-N	N-Iso
**cpd 1**	**0**	**1**	**0%**	**100%**	**138.5**	**85.6**	**189**
6.1	1.25	6.89	16%	84%	145.7	84.7	170.2
6.2	0.89	3.3	22%	78%	146.0	86.6	172.0
6.3	1.48	3.47	31%	69%	146.1	89.3	167.4
6.4	1.88	2.9	40%	60%	146.0	93.5	162.6
6.5	1.09	1.36	45%	55%	146.6	92.9	162.4
6.6	3.76	2.96	57%	43%	139.9	95.3	155.9
6.7	3.96	1.42	74%	26%	141.9	99.9	152.4
6.8	5.36	0.83	87%	13%	144.0	106.7	151.0
**cpd 13**	**1**	**0**	**100%**	**0%**	**141.8**	**113.5**	**149.6**

Table SI-6: Masses (mg) weight percent (wt %) and transition temperatures (°C) for binary mixtures (6.**X**) of compounds **1** and **13**. Data plotted in the text as Figure 7a.

Sample No.	Masses [mg]		Weight percent		Transition Temperatures		
	cpd 14	cpd 1	cpd 14	cpd1	MP	Nx-N	N-Iso
**cpd 1**	**1**	**0**	**0%**	**100%**	**138.5**	**85.6**	**189**
7.1	3.87	0.46	10%	90%	132.1	-	176.7
7.2	8.3	1.7	16%	84%	125.0	-	179.0
7.3	2.03	1.65	24%	56%	105.0	-	186.3
7.4	2.92	0.96	31%	76%	114.8	-	186.4
7.5	2.22	1.06	44%	69%	101.7	-	190.9
7.6	4.5	5.5	55%	45%	102.0	-	192.0
7.7	3.4	6.6	66%	34%	103.0	-	195.5
7.8	1.47	5.76	79%	21%	104.7	-	197.2
7.9	0.8	4.04	83%	17%	108.8	-	199.3
**cpd 14**	**0**	**1**	**100%**	**0%**	**117.1**	**-**	**204.2**

Table SI-7: Masses (mg) weight percent (wt %) and transition temperatures (°C) for binary mixtures (7.**X**) of compounds **1** and **14**. Data plotted in the text as Figure 7b.

Sample No.	Masses [mg]		Weight percent		Transition Temperatures		
	cpd 15	cpd 1	cpd 15	cpd1	MP	Nx-N	N-Iso
**cpd 1**	**0**	**1**	**0%**	**100%**	**138.5**	**85.6**	**189**
8.1	0.17	6.72	3%	98%	127.0	80.75	179.9
8.2	0.34	6.55	5%	95%	126.4	79.89	175.0
8.3	0.52	6.38	8%	93%	121.8	77.54	176.3
8.4	0.69	6.21	10%	90%	122.0	74.62	178.3
8.5	1.03	5.86	15%	85%	118.9	-	175.4
8.6	1.38	5.52	20%	80%	118.2	-	173.7
8.7	1.72	5.17	25%	75%	107.8	-	167.3
8.8	2.41	4.48	35%	65%	111.0	-	171.5
8.9	3.45	3.45	50%	50%	123.4	-	166.7
8.10	4.28	2.62	62%	38%	129.0	-	168.0
8.11	5.17	1.72	75%	25%	135.6	-	166.2
8.12	5.59	1.31	81%	19%	142.0	-	164.0
**cpd 15**	**0**	**1**	**100%**	**0%**	**151.6**	**-**	**165.9**

Table SI-8: Masses (mg) weight percent (wt %) and transition temperatures (°C) for binary mixtures (8.**X**) of compounds **1** and **15**. Data plotted in the text as Figure 8.

Sample No.	Masses [mg] cpd 17	cpd 2	Weight percent cpd 17	Cpd2	Transition Temperatures MP	Nx-N	N-Iso
**cpd 2**	**0**	**1**	**0%**	**100%**	**139.8**	**132.7**	**187.9**
9.1	20.2	3.8	84%	16%	132.0	129.9	186.0
9.2	18.5	3.8	83%	17%	131.3	131.0	182.1
9.3	18	4.1	81%	19%	131.7	131.2	181.9
9.4	18.9	6.4	75%	25%	130.5	131.1	182.0
9.5	16.1	7.7	68%	32%	142.0	130.5	175.0
9.6	15.9	9.2	63%	37%	147.0	131.4	172.0
9.7	18	18.8	49%	51%	148.5	132.9	171.0
9.8	10.2	15.1	40%	60%	158.3	134.0	163.7
9.9	7.7	15.7	33%	67%	156.0	133.7	160.0
9.10	4.9	17.2	22%	78%	159.8	135.4	159.8
9.11	2.2	18	11%	89%	163.5	137.5	158.5
**cpd 17**	**0**	**1**	**100%**	**0%**	**165.2**	**139.6**	**155.2**

Table SI-9: Masses (mg) weight percent (wt %) and transition temperatures (°C) for binary mixtures (9.**X**) of compounds **2** and **17**. Data plotted in the text as Figure 8.
